# HPV-Related Prognostic Signature Predicts Survival in Head and Neck Squamous Cell Carcinoma

**DOI:** 10.1155/2022/7357566

**Published:** 2022-11-15

**Authors:** Hongyu Zhao, Fengxu Wang, Xuehai Wang, Xinyuan Zhao, Jinfeng Ji

**Affiliations:** ^1^Department of Radiotherapy Oncology, Affiliated Hospital of Nantong University, Nantong 226000, China; ^2^Department of Integrated Traditional Chinese and Western Internal Medicine, Affiliated Tumor Hospital of Nantong University, Nantong Tumor Hospital, Nantong 226631, China; ^3^Department of Occupational Medicine and Environmental Toxicology, Nantong Key Laboratory of Environmental Toxicology, School of Public Health, Nantong University, Nantong 226019, China

## Abstract

**Background:**

Head and neck squamous cell carcinoma (HNSCC) is one of the most common cancers, worldwide. Considering the role of human papilloma virus (HPV) in tumor development and sensitivity to treatment of HNSCC, we aimed to explore the prognostic classification ability of HPV-related signatures in head and neck cancer.

**Methods:**

HPV-related signatures were screened out based on Gene Expression Omnibus (GEO) and the Cancer Genome Atlas (TCGA) databases. HPV-related signatures with prognostic value were identified through univariate Cox regression analysis and a risk signature was established by least absolute shrinkage and selection operator (LASSO). Further, we developed a nomogram by integrating independent prognostic factors.

**Results:**

A total of 55 HPV-associated signatures were differentially expressed and ten of them were associated with prognosis of HNSCC patients. The prognostic signature based on CDKN2A, CELSR3, DMRTA2, SERPINE1, TJP3, FADD, and IGF2BP2 expression was constructed. Univariate and multivariate regression analyses demonstrated that the novel prognostic signature was an independent prognostic factor of HNSCC. The nomogram integrating the prognostic signature and other independent prognostic factors was developed.

**Conclusion:**

In summary, the prognostic signature of the HPV-related signatures might serve as an important prognostic biomarker for patients with HNSCC.

## 1. Introduction

Nearly 95% of head and neck cancer cases are caused by head and neck squamous cell carcinoma (HNSCC), considered the sixth most common form of cancer globally [1]. It has been reported that, despite advances in treatment modalities, outcomes of HNSCC patients have not been improved significantly [2]. Local invasion and distant metastasis at first diagnosis are the main reasons for their worse prognosis [1]. Therefore, it is urgent to construct new prognostic models to aid early clinical diagnosis.

As far as etiology is concerned, HNSCC is primarily caused by tobacco and alcohol use [3]. Throughout the world, research studies on HPV have proliferated in recent years [4]. Globally, approximately 25% of the number of head and neck cancer cases are caused by HPV infection [4]. Also, HPV causes almost all cases of cervical cancer and a subset of other anogenital cancers [5].

In this study, we tried to identify HPV-related signatures and construct a novel prognostic model and nomogram by using sequencing data and clinically relevant information of patients with HNSCC. We expect to improve the diagnosis and prognosis of patients with head and neck cancer from the perspective of HPV.

## 2. Materials and Methods

### 2.1. Data Collection

To identify HPV-related signatures, we selected and downloaded GSE65858 cohort from Gene Expression Omnibus (GEO, https://www.ncbi.nlm.nih.gov/geo/) [6], which included 73 HPV-positive and 196 HPV-negative patients with HNSCC, and clinical information is shown in Supplementary Table 2. The package “limma” [7] was used for differential expression analysis with the screening criteria (adj.*p* < 0.05). Transcript expression profiling of 501 HNSCC tumor samples and 44 adjacent normal tissues was obtained from TCGA [8], and clinical information is shown in Supplementary Table 3.

### 2.2. Identification of Differentially Expressed HPV-Related Signatures

HPV-related signature expression in HNSCC patients was analyzed by using the “limma” package [7], with selection standard of |log (FC)| ≥ 2 and adj.*p* < 0.01.

### 2.3. Enrichment Analysis

To reveal the biological features of hub HPV-related signatures, Gene Ontology (GO) [9] and Kyoto Encyclopedia of Genes and Genomes (KEGG) [10] pathway analyses were conducted in this part. Tightly linked interaction network may facilitate the exploration of specific mechanisms in HNSCC, and closely linked genes may lead to an accurate prognostic model. In parallel, we constructed a protein interaction network by using the STRING database (https://string-db.org/) [11] in order to visualize the hub signatures.

### 2.4. Construction and Verification of the Prognostic Signature

Prognosis-associated signatures were picked out through the univariate Cox regression analysis with *p* < 0.01 in TCGA cohort. To avoid overfitting, least absolute shrinkage and selection operator (LASSO) analysis was applied to further screen HPV-related signatures. We calculated the risk score for each HNSCC patient by using the following formula: Risk score = gene (A) expression × coef (A) + gene (B) expression × coef (B) + gene (i) expression × coef (i) [12]. Patients with HNSCC were divided into the high- and low-risk groups according to the median risk score. Kaplan–Meier survival analysis was used to compare the overall survival rate between the high- and low-risk groups. Principal component analysis (PCA) was used to evaluate the clustering ability of the prognostic signature.

### 2.5. Immune Infiltration Analysis

In this study, we attempted to perform immune infiltration analysis by using the CIBERSORT [13] and single-sample Gene Set Enrichment Analysis (ssGSEA) algorithm [14]. Moreover, we attempted to mine drugs targeting the high-risk group with poor prognosis by using the Genomics of Drug Sensitivity in Cancer (GDSC) database [15].

### 2.6. Cox Regression Analyses and Construction of a Nomogram

Univariate and multivariate Cox regression analyses were used to evaluate independent prognostic factors in HNSCC. A nomogram integrating the independent prognostic factors was established by “rms” package [16]. Calibration curves and area under the curve (AUC) were used to verify the validity of the nomogram [17, 18] we constructed.

## 3. Results

### 3.1. Expression and Pathway Enrichment of HPV-Related Signatures in HNSCC Patients

**Figure 1:**
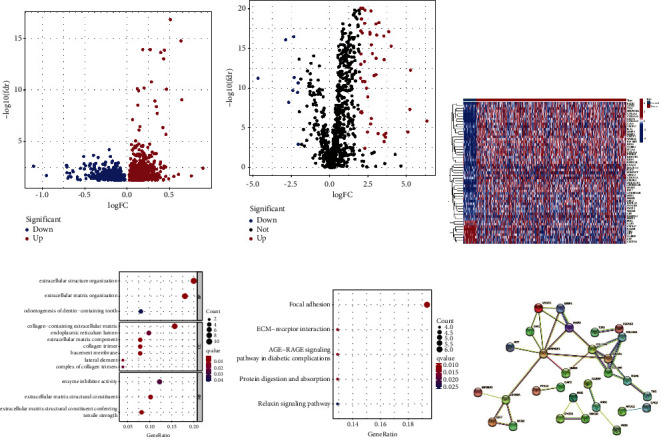
Expression and pathway enrichment of HPV-related signatures in HNSCC patients. (a) Volcano plot of HPV-associated signatures, red dots represent upregulated and blue dots represent downregulated genes; (b) volcano plot of TCGA cohort, red dots represent upregulated and blue dots represent downregulated genes; (c) heat map of TCGA cohort, the horizontal axis represents HNSCC samples and the vertical axis represents hub genes; (d) GO terms of HPV-associated signatures; (e) KEGG pathways of HPV-associated signatures; and (f) PPI network of HPV-associated signatures, the thicker the lines, the tighter the relationships.

We derived 1136 HPV-associated signatures with significant differences from the GSE65858 cohort (Figure 1(a), Supplemental Table 1). A total of 55 HPV-associated signatures were differentially expressed in TCGA cohort according to screening criteria (Figures 1(b)-1(c)). In the light of GO and KEGG pathway analyses, HPV-associated signatures were significantly accumulated in focal adhesion, ECM-receptor interaction, AGE-RAGE signaling pathway in diabetic complications, protein digestion and absorption, and relaxin signaling pathway (Figures 1(d)–1(e)). Also, a protein interaction network was constructed with tightly linked HPV-associated signatures (Figure 1(f)).

### 3.2. Construction and Validation of a Prognostic Model Based on HPV-Related Signatures in HNSCC Patients

**Figure 2:**
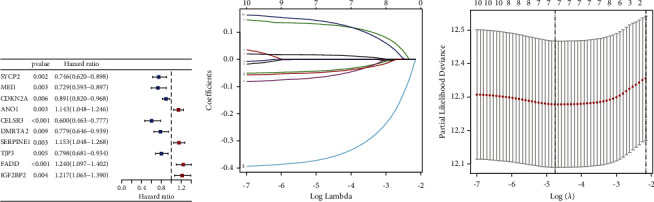
Construction of a prognostic signature based on HPV-related signatures. (a) Univariate Cox regression analysis, blue dots represent protective factors and red dots represent risk factors; (b) LASSO coefficient profiles, curves represent prognostic HPV-related signatures screened from univariate Cox regression analysis; and (c) LASSO deviance profiles.

Ten prognosis-related signatures were identified by univariate Cox regression analysis (Figure 2(a)). To avoid overfitting, LASSO regression was performed and selected seven HPV-related signatures for constructing the prognostic signature (Figures 2(b)-2(c)). Risk score = (−0.0413836017920187 × Exp CDKN2A) + (−0.365345241419471 × Exp CELSR3) + (−0.0633132503419892 × Exp DMRTA2) + (0.0162329920245241 × Exp SERPINE1) + (−0.0468942153601447 × Exp TJP3) + (0.12874495442016 × Exp FADD) + (0.139702586686539 × Exp IGF2BP2).

**Figure 3:**
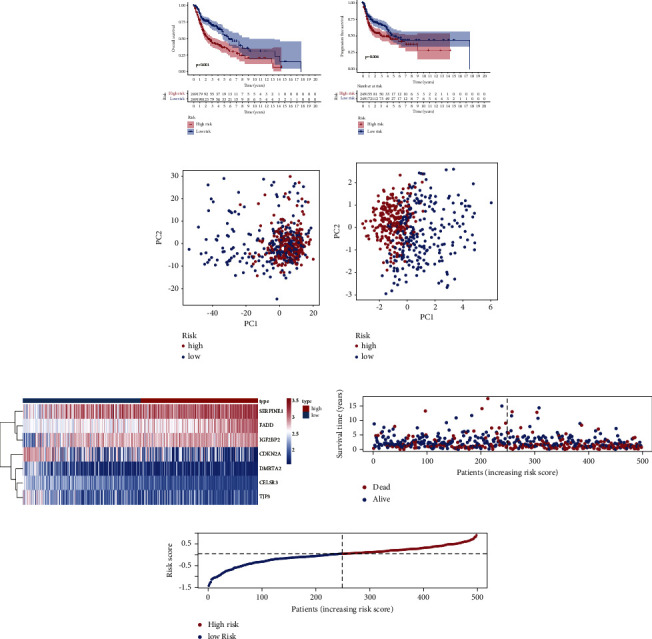
Validation of the prognostic signature. (a) Kaplan–Meier curve for overall survival, the blue curve represents the low-risk group and red curve represents the high-risk group; (b) Kaplan–Meier curve for progression-free survival, the blue curve represents the low-risk group and red curve represents the high-risk group; (c) principal component analysis without prognostic signature, the blue dots represent patients in the low-risk group and red dots present patients in the high-risk group; (d) principal component analysis with prognostic signature, the blue dots represent patients in the low-risk group and red dots present patients in the high-risk group; (e) heat map of HPV-related signatures expression; (f) survival status plot based on prognostic signature; and (g) risk score plot classified by prognostic signature.

**Figure 4:**
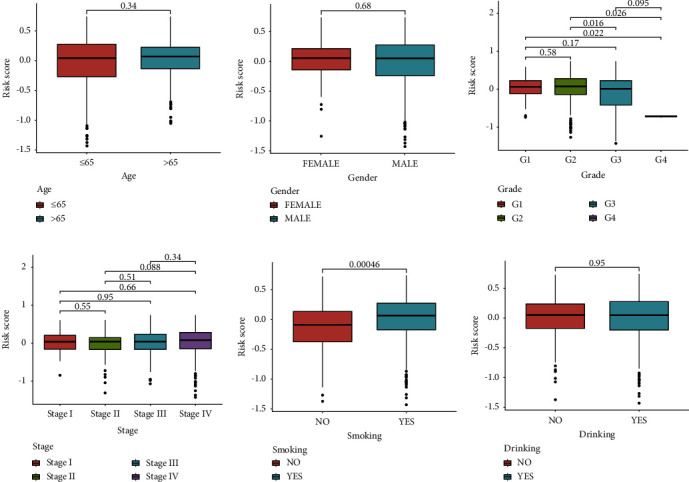
Relationship between prognostic signature expression and age (a), gender (b), grade (c), stage (d), smoking status (e), and drinking (f).

Kaplan–Meier survival curves showed a worse overall survival and progression-free survival in the high-risk group when compared with the low-risk group (Figures 3(a)-3(b)). Meanwhile, the prognostic signature showed a great clustering ability compared with the cohort without genotyping (Figures 3(c)-3(d)). As shown in Figure 3(e), seven HPV-related signatures are ranked in different groups. The distribution of the risk scores and survival time are shown in Figures 3(f)-3(g). Meanwhile, the time-dependent ROC curves of the prognostic signature were performed and shown in Supplemental Figure 1A. The relationship between prognostic signature and clinicopathological factors is shown in Figures 4(a)-4(f). Interestingly, we found that the risk score was highly expressed in smokers compared to nonsmokers (*p* = 0.00046, Figure 4(e)). In addition, HPV-negative patients had a significantly higher risk value than HPV-positive patients with HNSCC (Supplemental Figure 1B).

### 3.3. Immune Infiltration Analysis

**Figure 5:**
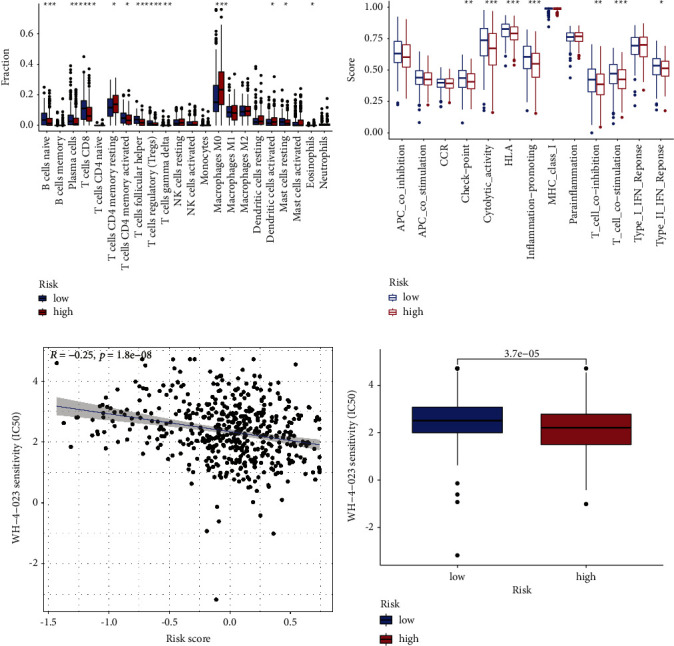
Relationship between prognostic signature and immune infiltration. (a) The infiltrating levels of 22 immune cells in low- and high-risk groups via CIBERSORT algorithm; (b) the levels of immune activity in low- and high-risk groups via the ssGSEA algorithm; and (c)-(d) relationship between WH-4-023 sensitivity and risk scores.

Through CIBERSORT algorithm, we found that naive B cells, plasma cells, CD8 T cells, activated memory CD4 T cells, follicular helper T cells, regulatory T cells (Tregs), gamma delta T cells, and resting mast cells were highly enriched in the low-risk group (Figure 5(a)) while resting memory CD4 T cells, M0 macrophages, activated dendritic cells, and eosinophils were highly enriched in the high-risk group (Figure 5(a)). Based on the ssGSEA algorithm, moreover, check-point, cytolytic-activity, HLA, inflammation-promoting, T cell co-inhibition, T cell co-stimulation, and type II IFN response were highly enriched in the low-risk group (Figure 5(b)). Moreover, WH-4-023, an inhibitor of LCK/SRC, was found to be useful in the high-risk group through pRRophetic algorithm (Figures 5(c)-5(d)).

### 3.4. The Prognostic Signature Was an Independent Prognostic Factor for HNSCC Patients

**Figure 6:**
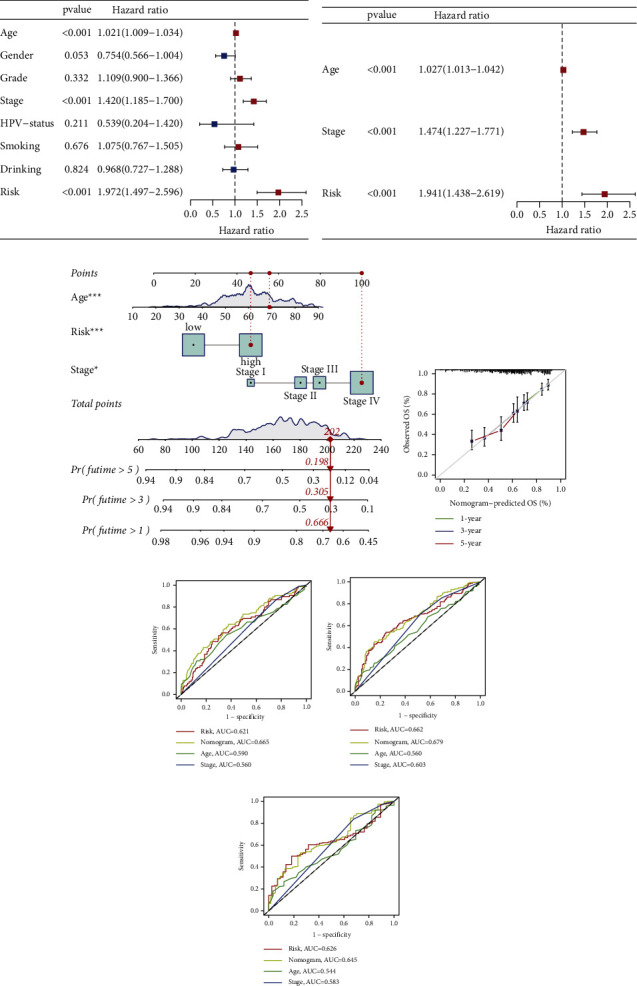
Independent prognostic analysis and construction of a novel nomogram. (a) Univariate Cox regression analysis for the overall survival of HNSCC patients; (b) multivariate Cox regression analysis for the overall survival of HNSCC patients; (c) the nomogram on the basis of independent prognostic factors; (d) time-dependent calibration curves of the nomogram; (e) ROC curves for one-year OS; (f) ROC curves for three-year OS; and (g) ROC curves for five-year OS.

Using both univariate and multivariate Cox regression analyses, age, stage, and the prognostic signature were found to be reliable independent predictors of HNSCC (Figures 6(a)-6(b)). Subsequently, a nomogram integrating independent prognostic factors with significant differences was developed to predict the one-, three-, and five- year OS of HNSCC patients (Figure 6(c)). In calibration curves, the predictive curves originated from the nomogram showed a high agreement with the ideal curve (Figure 6(d)). AUC values of the nomogram were 0.665, 0.679, and 0.645 for one-, three-, and five-year OS, respectively, demonstrating a better predictive accuracy of the nomogram compared with that of a single clinical factor (Figures 6(e)–6(g)).

## 4. Discussion

Head and neck squamous cell carcinoma is a collection of epithelial tumors originating mainly from the mucosa of the oral cavity, oropharynx, larynx, or hypopharynx [19]. Despite continuous advances in treatment modalities, the prognosis of patients with head and neck cancer has not improved significantly over the past decades [20]. The high recurrence rate of patients and the low response to intervention treatments such as chemoradiotherapy are the main reasons for the poor prognosis of patients [21, 22].

In recent years, the popularization and rapid development of next-generation sequencing (NGS) technology have enabled us to have a new understanding and insight into the molecular landscape of different tumors [23]. For head and neck cancer, these advances provided new insights into the different molecular mechanisms of HNSCC, the tumor microenvironment (TME) and heterogeneity of HNSCC, and the diversity of clinical responses among HNSCC subtypes [23–25].

**Figure 7:**
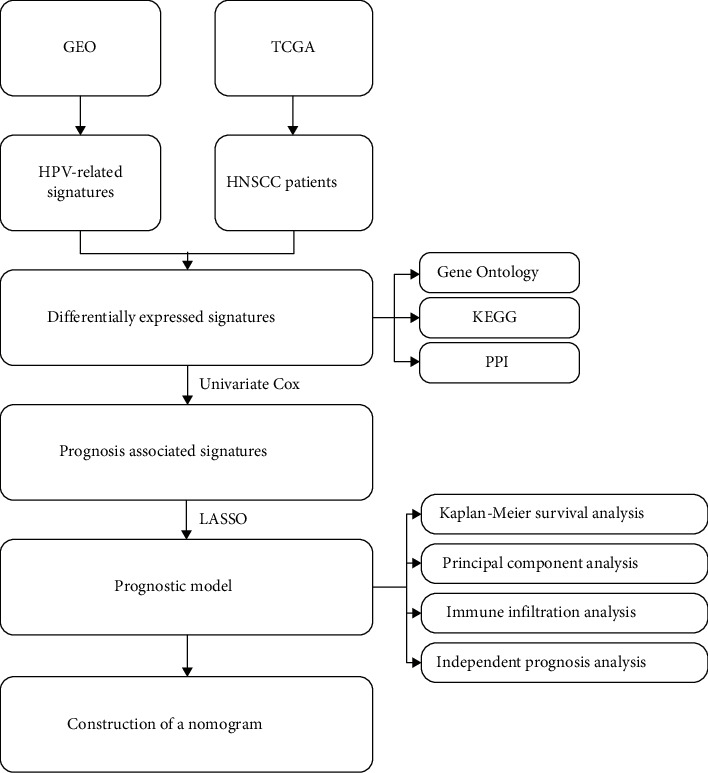
Flowchart of this study.

In this study, we attempted to construct a new prognostic model from the perspective of HPV by microarray and high-throughput sequencing data. The flowchart of this study is shown in Figure 7. On the basis of the important impact of HPV on HNSCC, we identified HPV-associated signatures by comparing the transcriptome data of HPV-positive and HPV-negative HNSCC patients from the GEO dataset. To achieve a larger sample size to justify the scientific quality of this research, TCGA database was used for subsequent analyses. Ultimately, 55 HPV-associated signatures were differentially expressed and were significantly accumulated in focal adhesion, ECM-receptor interaction, AGE-RAGE signaling pathway in diabetic complications, protein digestion and absorption, and relaxin signaling pathway. Subsequently, seven HPV-related signatures (CDKN2A, CELSR3, DMRTA2, SERPINE1, TJP3, FADD, and IGF2BP2) that enabled the classification of high- and low-risk HNSCC patients were screened out.

Among these prognostic HPV-associated signatures, we noted one of the interesting. Cyclin-dependent kinase inhibitor 2A, also known as p16, plays a critical role in cell-cycle regulation [26]. In addition, infection with HPV leads to overexpression of p16, and thus p16 is often used as a common marker of HPV positivity [26, 27]. Recently, multiple studies have surfaced that p16 expression in head and neck cancers was independent of HPV infection and should not serve as a reliable marker for HPV infection [28, 29]. Consistent with previous studies, p16 functioned as a tumor suppressor in HNSCC patients of this study and as a risk score reducer in the prognostic model with a negative coef value.

Interestingly, in the present study, we found that smoking HNSCC patients had a higher risk value. These results indicated that smoking would affect HPV status as well as the expression of HPV-related signatures and may give clues to the mechanism of head and neck cancers. Through prognostic analysis, we validated the prognostic model's validity and accuracy. In addition, immune infiltration analysis showed a higher level of immune cell infiltration in the low-risk group than that of the high-risk group, which may explain the worse prognosis in the high-risk group. To our knowledge, this study is the first attempt to construct a prognostic signature for head and neck cancer based on HPV-related genes. For the convenience of clinical application, a novel nomogram based on independent prognostic factors of head and neck cancer was constructed.

Meanwhile, in our study, we encountered a few limitations. The sample size and cohort size need to be expanded to guarantee their accuracy for this study. More patients with HNSCC and more prospective clinical trials need to be included in the calculation of the prognostic model for reducing statistical bias. Moreover, further biochemical experiments in vivo and in vitro on the seven HPV-related signatures should be conducted further.

## 5. Conclusion

We screened out differentially expressed signatures between HPV^+^ and HPV^−^ HNSCC patients and developed a novel prognostic signature based on large sample datasets. Meanwhile, a novel nomogram was constructed by integrating independent prognostic factors.

## Data Availability

The datasets analyzed during the current study are available in TCGA repository (https://portal.gdc.cancer.gov) and GEO (https://www.ncbi.nlm.nih.gov/geo/).

## Supplementary Materials

Supplementary Materials Supplemental Table 1: HPV-associated signatures with significant differences from the GSE65858 cohort. Supplemental Table 2: clinical information of the GEO cohort. Supplemental Table 3: clinical information of TCGA cohort. Supplemental Figure 1: ROC curves of the prognostic signature (A); the relationship between the prognostic signature and HPV status (B).
