# Supplementary material for: HPV-Related Prognostic Signature Predicts Survival in Head and Neck Squamous Cell Carcinoma
Source: J Oncol. 2022 Nov 15;2022:7357566. doi: 10.1155/2022/7357566 (PMC9681561; doi:10.1155/2022/7357566)
Supplement: Supplementary Materials — Supplemental Table 1: HPV-associated signatures with significant differences from the GSE65858 cohort. Supplemental Table 2: clinical information of the GEO cohort. Supplemental Table 3: clinical information of TCGA cohort. Supplemental Figure 1: ROC curves of the prognostic signature (A); the relationship between the prognostic signature and HPV status (B). [file 7357566.f1.zip › Supplemental Table 1 (1).pdf]

Supplemental Table 1: HPV-associated signatures with significant differences from the GSE6

ID	logFC	AveExpr	t	P.Value	adj.P.Val
SPRR2G	-1.105550505	9.196647442	-4.409179214	1.50E-05	0.002304779
KRTDAP	-0.952230934	10.5108414	-3.545743948	0.000461712	0.01839659
TMEM16	-0.716720468	9.168749421	-4.478231493	1.11E-05	0.001919325
ANO1	-0.713561082	8.806991904	-4.473054991	1.14E-05	0.001940337
PTHLH	-0.711217508	8.591499433	-4.340251983	2.02E-05	0.002752864
GJB6	-0.711064964	9.966285637	-3.837648798	0.000154911	0.009615422
KRT1	-0.699769285	7.957557179	-3.253782896	0.001284544	0.033167208
SPRR2B	-0.690117193	8.219047019	-3.419930252	0.000723723	0.024021704
DSG1	-0.676298671	8.090041227	-3.780597578	0.000192803	0.011129992
MMP3	-0.664068998	9.234131656	-3.393466978	0.000794177	0.025327224
CXCL14	-0.643352549	9.058984907	-4.013745145	7.76E-05	0.0063791
IGFBP6	-0.635242067	8.823435819	-3.916488535	0.000114	0.008011159
MMP1	-0.587965339	9.613117977	-3.172345125	0.001687668	0.038749359
KRT16	-0.587455654	11.08367575	-3.383964247	0.000821002	0.025661285
KRT14	-0.58063643	11.85189185	-3.106056015	0.002099038	0.044519458
LOC6513	-0.55325349	11.84235409	-3.07417973	0.002328157	0.047148931
TNC	-0.551581184	9.857801785	-4.218859819	3.36E-05	0.003729274
COL4A6	-0.512482274	7.59873296	-5.076352759	7.20E-07	0.000270375
CAV1	-0.508751397	9.289790255	-4.61721291	6.03E-06	0.001319697
CCND1	-0.503545677	11.12292405	-4.178518391	3.97E-05	0.004195649
SLC7A5	-0.489499465	9.918143	-4.449352605	1.26E-05	0.002059161
SERPINE	-0.488769632	8.370756952	-3.601590724	0.000376649	0.016416306
GJB2	-0.484987282	11.75240634	-3.204281685	0.001517345	0.036358362
IGFBP3	-0.450193437	9.63411701	-3.533959	0.000481829	0.018822333
NT5E	-0.442639789	7.834106549	-4.477342461	1.12E-05	0.001919325
F2RL1	-0.434450297	8.150277801	-4.614303645	6.11E-06	0.001319697
GJA1	-0.433596355	10.47943929	-3.386074348	0.000814974	0.025545532
DKK3	-0.42724983	10.04142932	-4.201663176	3.61E-05	0.00392592
SPINK6	-0.421344389	7.055107	-3.225466358	0.001413301	0.034868751
KRT17	-0.419038837	12.66982538	-3.823708531	0.000163458	0.00997051
PPFIA1	-0.408478636	8.268053954	-4.132597397	4.80E-05	0.004622417
DMKN	-0.403860969	8.432069733	-3.34208143	0.000949616	0.027858358
TUBB2A	-0.4037235	8.733781685	-3.560973059	0.000436882	0.017863091
THBS1	-0.403063861	8.662558433	-3.225336267	0.00141392	0.034868751
FKBP9L	-0.397216308	9.898603477	-5.152263476	4.99E-07	0.000207078
HSPC159	-0.393462758	8.392172363	-3.673859253	0.000288319	0.014268537
COL4A1	-0.393060702	10.23279949	-3.561634379	0.000435833	0.017863091
FADD	-0.390443733	8.945675492	-3.485173106	0.000574177	0.020811935
CDK6	-0.390339347	8.79496705	-4.553065689	8.02E-06	0.001546141
SESN3	-0.388379168	7.631070369	-4.354699661	1.90E-05	0.002688854
CTTN	-0.385618447	8.601521973	-4.40626425	1.52E-05	0.002304779
RGS20	-0.378894046	7.537924021	-4.133647859	4.78E-05	0.004622417
GSTA4	-0.378688123	8.731373448	-3.220852226	0.001435383	0.035185616
NIPAL4	-0.375558513	8.032800577	-3.390025329	0.000803797	0.025419163
DLL1	-0.369625718	8.5547989	-4.658381082	5.02E-06	0.001185747
PPP1R14C	-0.36729574	8.413379662	-3.942667737	0.000102851	0.007621426
ORAOV1	-0.366673245	8.175908855	-3.396189331	0.000786645	0.025223725
TUBB3	-0.365196705	8.174215609	-3.517496395	0.00051131	0.019404175
PYGL	-0.353086056	9.824612167	-3.218697294	0.001445804	0.035337772

IRX2	-0.349958063	7.876394485	-3.869700077	0.000136836	0.008902581
CD36	-0.348282505	7.302059807	-3.950112088	9.99E-05	0.007489916
MYH10	-0.346527123	9.53779027	-3.491882555	0.00056056	0.020548
MAGEA4	-0.346139844	7.017378543	-3.05259784	0.002496126	0.0491024
HTRA1	-0.345751245	9.068318669	-3.524401672	0.000498744	0.019210951
DSC1	-0.345718216	6.986964185	-3.228375828	0.00139954	0.034748138
PRSS23	-0.345447256	8.838320513	-3.756843452	0.000211032	0.011709633
FAM89A	-0.342920927	8.154854944	-5.011677821	9.80E-07	0.000331281
KRT17P3	-0.341661203	8.548085768	-3.404674309	0.000763592	0.024873935
DHCR7	-0.340750813	8.383881835	-3.106449791	0.002096342	0.044505244
TMEM16(	-0.338345452	7.62485167	-3.223256213	0.001423839	0.035014549
TNFRSF1.	-0.337899548	8.289832595	-3.276538344	0.001189058	0.031707744
STC1	-0.330613137	7.861302632	-3.493503991	0.000557315	0.02047808
ME1	-0.329337595	8.340678654	-3.241441438	0.001339262	0.033976898
MYO1B	-0.329335662	9.116163022	-4.239647412	3.08E-05	0.003563901
SPOCK1	-0.328379376	7.286015	-3.723732608	0.000239172	0.012694051
WDR66	-0.32755167	8.981388676	-3.275948425	0.001191448	0.03173296
IGF2BP2	-0.326148238	9.464010419	-3.050532638	0.00251277	0.04925344
RHOB	-0.322889153	10.11986552	-3.421487271	0.000719765	0.023994813
FSCN1	-0.321894759	11.77891728	-4.174770213	4.03E-05	0.004215145
CDA	-0.320303788	7.514906838	-3.17050142	0.001698022	0.038824799
EFNB2	-0.317847345	8.989719418	-3.685421963	0.000276143	0.013980842
IL24	-0.317783796	7.473982488	-3.121122031	0.00199815	0.043086705
ZFP36	-0.310056167	10.84934567	-3.323412209	0.001012791	0.028850133
SNAI2	-0.300763446	8.612829659	-3.422790505	0.000716468	0.023932065
SULF2	-0.296163892	8.590051766	-3.554047421	0.000448015	0.018129329
VEGFC	-0.293860435	7.370367173	-3.193706998	0.001571894	0.037168131
CDH13	-0.292922728	7.508853696	-3.86508817	0.000139307	0.008902581
DUSP14	-0.29198077	9.205883494	-3.722250598	0.000240511	0.012734349
PRNP	-0.289105611	10.72758798	-3.796411553	0.000181504	0.010778865
TCHH	-0.287387721	6.946650248	-3.189545925	0.001593852	0.037456376
CAV2	-0.286832418	8.425404105	-3.612005149	0.000362513	0.016190049
RNF217	-0.285600708	7.464424334	-4.590249553	6.80E-06	0.001419142
LTBR	-0.278881473	9.45863309	-4.18588017	3.85E-05	0.004170266
BTBD11	-0.277098605	8.452520133	-3.108936113	0.002079396	0.0442738
HOXC13	-0.274365882	7.402588929	-3.782608092	0.000191331	0.01109263
RRAS2	-0.274213946	8.099666983	-4.138090383	4.69E-05	0.00460036
PNLIPRP:	-0.273962649	6.812076704	-3.274791843	0.001196147	0.031802072
FAM83B	-0.273798677	8.030679012	-3.355877069	0.000905312	0.02720647
CEBPB	-0.273187742	11.2043226	-3.752145186	0.000214826	0.011890084
HSPH1	-0.269947702	10.51062014	-3.942395208	0.000102961	0.007621426
ANKRD5'	-0.269728844	10.66783482	-3.237736107	0.001356107	0.033976898
MAGEA9.	-0.269155924	6.768468529	-3.602922778	0.000374812	0.016416306
MN1	-0.268128619	7.748299541	-3.483910573	0.000576774	0.020811935
DYNC1I1	-0.267172009	6.996447911	-3.380338315	0.000831459	0.025841083
RGS2	-0.264877811	10.00901439	-3.078795862	0.002293609	0.046803621
TGFA	-0.264097374	8.431403865	-3.355548158	0.000906346	0.02720647
SIRPA	-0.262046215	8.340728397	-4.153048029	4.41E-05	0.004445734
COL28A1	-0.261832783	6.790866793	-4.52802925	8.96E-06	0.001682113
CAP2	-0.261760128	7.663679959	-3.792056847	0.000184551	0.01084104
CUTL1	-0.259728175	8.95675171	-3.246598276	0.00131614	0.033588331

TPCN2	-0.258947735	7.61911771	-3.330859574	0.000987135	0.028420089
ZDHHC8	-0.258013327	10.00326304	-4.57733402	7.21E-06	0.00147403
CD93	-0.256954915	8.394663972	-3.135659926	0.001905059	0.041818053
CCDC86	-0.254637187	8.167275352	-4.734107243	3.56E-06	0.000943445
GNG11	-0.253837047	8.058470587	-3.425655776	0.000709269	0.023829912
PPP2R2C	-0.253268049	7.627240911	-3.246883458	0.001314873	0.033588331
IRS1	-0.252102129	7.493070221	-3.51542815	0.000515131	0.01951548
CCRN4L	-0.251432472	7.78933416	-3.430938207	0.000696173	0.023533849
FJX1	-0.246422819	7.885584997	-3.461643033	0.000624417	0.022105331
EFEMP1	-0.245872553	8.012477648	-3.164414352	0.001732622	0.039207944
CTSL1	-0.245359405	8.588260008	-3.342842054	0.000947122	0.027822328
KDELR2	-0.244666678	9.494608299	-4.063011405	6.36E-05	0.005548939
OAF	-0.243880386	8.368606444	-3.780346164	0.000192988	0.011129992
INHBB	-0.240767384	7.145504853	-3.62285888	0.000348313	0.015940668
SEC61G	-0.240092127	11.06831089	-3.663836779	0.00029928	0.014442048
SSFA2	-0.239548559	7.627294454	-3.892146927	0.000125387	0.008503463
LAMB1	-0.234158896	8.575643406	-3.17789568	0.001656851	0.03832208
MYO5A	-0.232950678	7.640675278	-4.027415749	7.34E-05	0.006206115
GALNTL4	-0.231174279	7.995138642	-3.680018429	0.000281771	0.014135531
PI15	-0.230616705	7.142506447	-3.376693442	0.000842095	0.025983148
PROCR	-0.229715517	7.532917548	-3.822205302	0.000164406	0.009979277
PAPPA	-0.228251448	7.302799322	-3.739468515	0.000225384	0.012197943
FMNL2	-0.227829661	7.998298022	-3.987260042	8.62E-05	0.006838213
TFAP2C	-0.225032706	8.717877569	-3.042088941	0.002581889	0.049940008
AGPAT4	-0.224016587	7.46770625	-3.763956516	0.00020541	0.011543395
GPR116	-0.223040522	8.259783965	-3.070842827	0.002353428	0.047485658
EML1	-0.21966413	7.3895142	-3.997880261	8.26E-05	0.006675624
DEGS1	-0.219256525	9.073646059	-3.376342829	0.000843125	0.025983148
BCHE	-0.217842452	6.810718695	-3.16618629	0.001722484	0.03909931
SLC3A2	-0.21756295	8.060027301	-4.303957336	2.35E-05	0.003005234
ITGA6	-0.216145063	7.759039226	-3.462613511	0.000622266	0.022088935
SVEP1	-0.216073939	7.547325027	-3.189682179	0.001593129	0.037456376
TTYH3	-0.21561026	8.713913308	-3.407464558	0.00075615	0.024710055
NT5C3	-0.21109057	9.086876598	-3.919319729	0.000112741	0.00796546
LOC10013	-0.210630235	7.085330894	-5.487170125	9.44E-08	5.76E-05
KRT9	-0.209154065	6.939106116	-3.29614455	0.00111212	0.030469592
KLK9	-0.206882482	6.72540255	-3.101567923	0.002129988	0.044813576
LAMC3	-0.204968356	7.12591066	-3.595151397	0.000385647	0.016615315
COL12A1	-0.201631542	7.425453052	-3.662796541	0.00030044	0.014442048
PTPLA	-0.200721014	7.614952242	-3.227937823	0.001401604	0.034760098
TNFRSF1	-0.199823874	9.387796153	-4.073826651	6.09E-05	0.005375666
PTGS1	-0.198687605	7.515890849	-3.138693576	0.001886146	0.041527346
NAV1	-0.198626503	7.499172207	-3.608921521	0.000366646	0.016308318
LOC72855	-0.196085462	8.268315246	-5.074506044	7.26E-07	0.000270375
C10orf108	-0.193447032	6.916197775	-3.539780635	0.000471791	0.018611612
DHRS7	-0.192931389	9.629227027	-3.261176335	0.001252766	0.032614967
WDFY2	-0.19023837	7.824392468	-3.879402507	0.00013177	0.008741317
C16orf57	-0.189974043	8.482893319	-3.736469427	0.000227953	0.012266244
MPP6	-0.189854415	7.176045281	-3.878232775	0.000132372	0.008741317
CHD7	-0.189080817	8.326488778	-3.736445246	0.000227974	0.012266244
BZW2	-0.188335187	9.520302254	-3.758579803	0.000209647	0.011691789

RILPL1	-0.186991222	8.602785934	-4.035853352	7.10E-05	0.00602272
KTN1	-0.185529977	8.661247801	-3.644794159	0.000321197	0.015210482
UNC84A	-0.184257019	8.887839744	-3.398589414	0.000780059	0.025095509
KIF5B	-0.183837972	8.314861253	-3.851847896	0.000146641	0.00917989
STARD3N	-0.183347783	7.532243356	-3.799396242	0.000179443	0.01073549
AGPAT9	-0.182157294	7.315903592	-3.069264051	0.002365473	0.047641187
HSPA5	-0.181343715	8.736945854	-3.192248543	0.001579558	0.037200033
LOC65051	-0.180069987	7.469726749	-3.400936328	0.000773669	0.025040788
UPP1	-0.179694642	8.145051667	-3.119907918	0.002006111	0.043173639
STRN3	-0.179540977	8.532331618	-3.284855634	0.001155835	0.031123969
SPAG9	-0.178735865	7.994687664	-4.71333765	3.92E-06	0.00101237
EGFR	-0.177760275	7.231810325	-3.74098245	0.000224098	0.012181382
EIF3B	-0.176890057	9.639535092	-3.864443318	0.000139656	0.008902581
KCNQ4	-0.176637582	6.826285354	-3.613770592	0.000360167	0.016183947
TMED10F	-0.175526581	10.83859623	-3.223094525	0.001424612	0.035014549
TBC1D16	-0.175087282	7.598814482	-3.736042249	0.000228321	0.012266244
ZDHHC9	-0.174819387	8.682904895	-3.204520515	0.001516133	0.036358362
RFTN2	-0.174011135	7.126568717	-3.240421417	0.001343879	0.033976898
RRS1	-0.173741926	7.994413974	-3.704442721	0.000257161	0.013295545
MEMO1	-0.172956364	8.07321775	-4.801601436	2.62E-06	0.000723965
DRAP1	-0.172772943	10.55275511	-3.158391904	0.001767497	0.039914903
EBPL	-0.172729099	8.47901971	-3.174088783	0.001677931	0.038631606
RAB11FII	-0.172637762	7.738268455	-4.083148884	5.87E-05	0.005239691
PANX1	-0.172308171	7.470850201	-3.328016409	0.000996857	0.028483673
TMEM98	-0.172104496	7.58247977	-3.242362975	0.001335102	0.033953941
HSP90AB	-0.171027592	10.91093441	-3.245137548	0.001322652	0.033715347
C3orf26	-0.170796134	8.214421309	-3.745822911	0.000220032	0.012086891
SBDS	-0.169144613	8.936130315	-3.488758509	0.000566862	0.020690478
SYNCRIP	-0.167180872	9.638218503	-3.187743372	0.001603452	0.037582513
GARS	-0.166545869	10.371292	-3.708114368	0.000253642	0.013144514
FOXF2	-0.166096014	7.004254394	-3.874171268	0.000134479	0.008847023
KHDC1L	-0.16583756	6.704043822	-3.794674158	0.000182714	0.010821481
TTPAL	-0.164029382	7.626004322	-3.714048966	0.000248049	0.012977109
NDEL1	-0.163874854	8.737067547	-3.193131552	0.001574914	0.037170332
GOLGA7I	-0.162665856	6.837523274	-4.072423522	6.13E-05	0.005384633
CSAG3A	-0.162236784	6.631446751	-3.934068122	0.000106394	0.007725808
DCUN1D:	-0.161936179	7.724127961	-3.710706769	0.000251184	0.013047931
C14orf37	-0.161710185	6.971564385	-3.391699145	0.000799105	0.025412709
LOC72968	-0.161340915	7.186328036	-3.201459039	0.001531731	0.036543679
DYNC1I2	-0.160727531	9.341089878	-3.635961872	0.000331867	0.015581457
DNM1L	-0.159948871	7.965264628	-4.09633571	5.56E-05	0.005048811
EXOSC6	-0.159329458	8.596889064	-3.276825153	0.001187898	0.031707744
RNF149	-0.159128382	8.9157657	-4.306662912	2.33E-05	0.002988454
LOC38966	-0.158177422	7.667818758	-3.438851256	0.000676977	0.023170118
HSPA12A	-0.157989981	7.070397751	-3.926234371	0.000109722	0.007853144
ZAK	-0.157933009	7.52643158	-3.69891614	0.000262546	0.013447362
NRP1	-0.156812426	7.488769415	-3.522681515	0.000501847	0.019210951
NSMAF	-0.156581007	8.855459615	-3.552553329	0.000450451	0.018156921
PGM3	-0.156484956	7.617006964	-4.100484225	5.47E-05	0.005043643
RAB2A	-0.156120174	8.374763906	-3.520782237	0.000505294	0.019233981
ACLY	-0.155173764	9.13130864	-3.763047151	0.000206121	0.011553794

SNX13	-0.154742761	7.756065544	-4.110601248	5.25E-05	0.004924133
TMEM181	-0.154534424	9.037400511	-3.786198357	0.000188727	0.010970649
STRAP	-0.15441971	10.25509243	-3.582708603	0.000403608	0.017087624
CAMSAP	-0.15302401	8.793035442	-3.054079502	0.002484247	0.048912517
STXBP5	-0.152561548	7.707384753	-3.112393347	0.002056041	0.044075511
PPPDE2	-0.152534257	8.009965535	-3.353097074	0.000914082	0.027325368
IDE	-0.151395688	7.811226841	-3.572696899	0.000418627	0.017454462
LMTK2	-0.149304324	7.074173945	-4.015933785	7.69E-05	0.0063791
COPS7A	-0.148682944	9.780920174	-3.607469168	0.000368608	0.016362452
SPRYD3	-0.147547902	8.032478071	-4.285486938	2.54E-05	0.003121601
SMOX	-0.146955713	7.158292372	-3.612195223	0.00036226	0.016190049
ZNF426	-0.145671072	7.011489829	-3.167974457	0.001712307	0.038960921
RSPH3	-0.145179912	7.841465364	-3.301404593	0.001092281	0.030141967
WDR43	-0.144751204	7.751577248	-3.826992445	0.000161406	0.009934354
PCDH21	-0.144438812	6.81151764	-3.226745051	0.001407238	0.034809879
TAX1BP1	-0.144102727	9.657852769	-3.969654045	9.24E-05	0.007127413
LAMP1	-0.142817422	10.99826507	-3.305591155	0.001076726	0.030023983
LOC72853	-0.142616653	8.888267434	-3.432100845	0.000693321	0.023473555
PFKM	-0.142413353	8.020296168	-3.314513399	0.001044261	0.02934214
GLG1	-0.142336685	10.12208003	-3.050082183	0.002516414	0.04927858
PCNX	-0.141797871	8.702655852	-3.077141021	0.002305939	0.046889602
OSMR	-0.141653952	7.061374851	-3.41743337	0.000730112	0.024160782
SNORA8	-0.141528688	7.077250655	-3.645706316	0.000320114	0.015191919
CSNK1D	-0.141505433	8.289021662	-3.550600145	0.000453654	0.018156921
C7orf30	-0.141439471	9.772457516	-3.669987484	0.000292508	0.01437869
EXT2	-0.140527997	7.688725727	-3.199569142	0.001541434	0.036695481
LOC64275	-0.140363476	8.672552999	-3.835237786	0.000156358	0.009677923
RPF2	-0.139367287	9.292711532	-3.170602363	0.001697453	0.038824799
MTDH	-0.138983526	9.788799828	-3.153249046	0.00179779	0.040226916
PWP1	-0.138835486	9.312020586	-4.481411526	1.10E-05	0.001915521
LOC10013	-0.138764876	7.614747391	-3.503927901	0.000536871	0.020096525
PDZD8	-0.138457337	7.693643476	-3.391262119	0.000800327	0.025412709
CYB5R3	-0.138119662	9.954494847	-3.42851278	0.000702157	0.0236997
AVL9	-0.137949117	7.02464855	-4.540225587	8.49E-06	0.001607981
PMM2	-0.137849158	8.14252565	-3.566120107	0.000428778	0.017643328
RSL1D1	-0.137796292	8.495656563	-3.069300861	0.002365191	0.047641187
LOC65329	-0.137760452	6.73508634	-3.521136926	0.000504649	0.019233981
SBDSP	-0.136852837	7.79941791	-3.298499369	0.001103197	0.030338614
LTV1	-0.136688588	8.898979086	-3.774836935	0.000197082	0.011257239
GFPT1	-0.136559652	8.477980944	-3.074700997	0.002324232	0.047112864
LOC72992	-0.136533402	6.770395145	-3.859756611	0.000142218	0.00897974
KLHL36	-0.135634951	7.988256756	-3.585044989	0.000400176	0.016998119
CSNK2A2	-0.135538572	7.95491874	-3.943483172	0.000102521	0.007621426
GALNS	-0.134789074	7.162270281	-4.636581448	5.53E-06	0.001240812
POP1	-0.134772649	7.109780421	-3.699242947	0.000262224	0.013447362
RUNDC3A	-0.134249772	6.597639578	-3.1513829	0.0018089	0.040311317
TOP1MT	-0.134156923	7.313198977	-3.79332602	0.000183658	0.010830997
PNO1	-0.134098159	8.020487095	-3.38753618	0.000810822	0.0254881
C16orf87	-0.133838561	7.570299403	-3.938959297	0.000104365	0.007621426
TMED2	-0.133718131	10.25661344	-3.338930981	0.000960013	0.028051008
UBE2F	-0.13371637	9.477481659	-3.049196248	0.002523595	0.049333595

ARIH1	-0.133037958	7.702627471	-3.662272267	0.000301027	0.014442048
GTF2F2	-0.132809943	9.339377962	-3.18246482	0.001631873	0.03792849
ZNF319	-0.132700204	7.650280611	-3.510994879	0.000523412	0.019795065
CSAG3B	-0.132372326	6.517534695	-3.454139397	0.000641284	0.022315256
E2F4	-0.130517378	8.814817842	-3.267744145	0.001225151	0.032169986
CHST2	-0.130180068	6.891227478	-3.301935063	0.001090298	0.030141967
HRSP12	-0.129210589	7.560764984	-3.171785079	0.001690807	0.038780903
RBMS1	-0.12915217	7.838410319	-3.540823432	0.000470014	0.018574851
PIP4K2C	-0.128653859	7.991842605	-3.401733273	0.00077151	0.025040461
FBXO28	-0.128520358	8.269103743	-3.23823076	0.001353847	0.033976898
GPATCH2	-0.128512051	7.32200569	-3.521503591	0.000503982	0.019233981
LOC64517	-0.128199893	7.469540188	-3.923243089	0.000111018	0.00787265
LOC28473	-0.128183645	6.587241621	-3.113658739	0.002047554	0.043936433
KLF10	-0.127962261	6.95338294	-3.295770281	0.001113544	0.030470622
FEZ2	-0.127813186	9.181709943	-3.23814757	0.001354227	0.033976898
TPD52L2	-0.127773388	8.988892526	-3.310724932	0.001057933	0.029688335
AUP1	-0.127722561	8.54324474	-3.476325173	0.000592607	0.021276729
UBR3	-0.127707685	7.713258995	-4.162141686	4.25E-05	0.004342387
ARCN1	-0.126820313	9.787091983	-3.26608438	0.001232075	0.032267455
KIF7	-0.125601255	6.787910984	-3.551936512	0.00045146	0.018156921
ZNF650	-0.125324319	7.627347842	-3.939149846	0.000104286	0.007621426
DDX10	-0.125005196	8.270948925	-3.065920758	0.002391166	0.047829454
NUPL2	-0.125004381	8.443335689	-3.18227037	0.001632929	0.03792849
LCLAT1	-0.124611273	8.088672064	-3.187575165	0.001604351	0.037582513
PEAR1	-0.124423338	6.78364864	-3.66752568	0.000295201	0.014414339
SMTN	-0.124250457	6.940702016	-3.317694255	0.001032909	0.029185086
SHC1	-0.123541297	8.276718833	-3.578439443	0.00040995	0.017289483
CCNC	-0.123411087	7.830992578	-3.057729516	0.002455206	0.048602022
C7orf42	-0.123379587	8.093596909	-3.491509979	0.000561308	0.020548
CREB5	-0.123164293	6.737927718	-4.588861248	6.85E-06	0.001419142
DOK4	-0.123130138	7.215240409	-3.099274154	0.002145968	0.044907949
RTN4	-0.122418929	8.442008387	-3.893519421	0.000124717	0.008499431
RALGAPI	-0.122249689	7.743555483	-3.637133255	0.000330433	0.015547346
PREP	-0.122133536	8.350533476	-3.099676668	0.002143155	0.044907949
GNA12	-0.121768371	7.14248533	-4.419804498	1.43E-05	0.002251854
TNS4	-0.121748792	6.931537381	-3.137066485	0.001896269	0.041708419
OSBPL5	-0.121674573	7.489279352	-3.192253599	0.001579531	0.037200033
DEXI	-0.121613298	8.685851439	-3.21331399	0.001472146	0.035838283
C14orf100	-0.120980739	8.187457022	-3.095780724	0.002170518	0.045177126
C14orf149	-0.120852075	7.00286407	-3.349087396	0.00092687	0.02748785
LOC10012	-0.120830678	7.12144495	-3.457732614	0.000633155	0.02220925
MRPL32	-0.120272187	9.661695458	-3.09597766	0.002169127	0.045177126
MRPL19	-0.119710841	8.602478456	-3.078009005	0.002299464	0.046826814
TMEM150	-0.119234353	7.597968551	-3.623458609	0.000347544	0.015940668
SNORA18	-0.119012263	6.992966243	-3.396944227	0.000784568	0.025203659
CD276	-0.118592788	7.42106852	-3.047451111	0.002537796	0.049444144
TAF1D	-0.118529223	7.299335163	-3.066558387	0.002386246	0.047829454
LOC64955	-0.118291249	8.379533393	-3.090331647	0.002209328	0.045596796
ETNK1	-0.118271679	7.268495499	-3.284298774	0.001158032	0.031144965
UBR1	-0.118000555	6.816741135	-3.965248229	9.41E-05	0.007227535
LOC65281	-0.117609652	6.734556245	-3.866724162	0.000138426	0.008902581

GTF2H3	-0.1175649	7.484982926	-3.395883264	0.000787488	0.025223725
CRIM1	-0.115954983	6.747459766	-4.611643481	6.19E-06	0.001319697
LRRC41	-0.115950947	8.412387198	-3.055969649	0.002469169	0.048746681
LOC2859?	-0.115792501	6.639433127	-3.793038049	0.00018386	0.010830997
ARFGEF2	-0.115505699	7.252785525	-3.846476804	0.000149719	0.009345944
SCYL1	-0.113755298	8.807302433	-3.273887484	0.001199832	0.031802072
ZNF419	-0.112832739	7.830443625	-3.249332059	0.001304034	0.033434699
TM4SF18	-0.112767967	6.881558278	-3.099816884	0.002142177	0.044907949
PIGT	-0.112735686	8.272163498	-3.047849573	0.002534547	0.049444144
NUDT15	-0.111600164	7.553211098	-3.522750317	0.000501723	0.019210951
CHORDC	-0.110886014	7.102244119	-3.659154713	0.000304535	0.014578521
TOR1AIP	-0.110503375	7.148026777	-3.259815462	0.00125856	0.032651514
LOC7292	-0.110393729	7.902055907	-3.213180269	0.001472806	0.035838283
LOC6530	-0.110380166	6.648473316	-3.574978278	0.000415159	0.017408967
ATF2	-0.108862103	7.083718488	-4.085085452	5.82E-05	0.005219946
AHR	-0.108229525	9.471173589	-3.148536447	0.001825969	0.040613722
THSD1	-0.108149445	6.705405448	-3.432352247	0.000692706	0.023473555
PES1	-0.107940139	7.526347721	-3.439657805	0.000675048	0.023140157
GGCX	-0.107934894	7.210428457	-3.825407576	0.000162393	0.009967223
LOC4002	-0.107345437	6.701662962	-3.664609377	0.000298422	0.014442048
TRIP11	-0.107274847	8.213099601	-3.07873447	0.002294065	0.046803621
SERINC3	-0.107153127	7.857129345	-3.829208793	0.000160034	0.0098776
GSPT1	-0.106768085	10.86161076	-3.128373413	0.001951205	0.042449329
TRIO	-0.106281612	7.134982959	-3.042829121	0.002575761	0.04986537
C1RL	-0.106023415	7.329156305	-3.427026367	0.000705849	0.023787757
SLC35F3	-0.105054575	6.567370829	-4.697016412	4.22E-06	0.001064955
FAM10A4	-0.103954686	8.308132309	-3.151692719	0.001807051	0.040310997
RBM9	-0.101783459	7.745663027	-3.394102081	0.000792414	0.025307721
STK31	-0.101632849	6.603593882	-3.44914799	0.000652739	0.022515919
PUS1	-0.101490962	8.223918728	-3.260804253	0.001254348	0.032617499
SELI	-0.099592111	7.406665424	-3.05485385	0.00247806	0.048834922
COPA	-0.099314403	9.787528225	-3.067279164	0.002380696	0.047829454
APH1B	-0.098395387	7.178288553	-3.078261976	0.00229758	0.046826814
METTL1	-0.097187156	7.936537303	-3.364512401	0.000878569	0.026661454
DLX1	-0.096646184	6.68729875	-3.622168365	0.000349201	0.015940668
TLL1	-0.095423088	6.627664966	-3.131998345	0.001928121	0.042113912
CSAG2	-0.094982855	6.41355672	-3.34841504	0.000929031	0.02748785
C2orf56	-0.09463645	7.45220866	-4.115354843	5.15E-05	0.004853234
AGAP3	-0.094354535	6.984944277	-3.67720369	0.000284746	0.014219827
TRAF3IP	-0.093354488	8.072161334	-3.054850857	0.002478084	0.048834922
SH3RF3	-0.093016308	6.734502061	-3.18059064	0.001642076	0.038060482
PTPN14	-0.092067606	6.697191786	-3.441983326	0.000669517	0.022986392
SRPK2	-0.091972688	6.930441856	-3.536597098	0.000477256	0.018759828
SUGT1	-0.091179953	7.474147463	-3.097227019	0.002160323	0.045122402
CYCSL1	-0.090988541	8.295752376	-3.203600843	0.001520803	0.03640154
KIAA142	-0.090342088	7.458546862	-3.254417146	0.001281789	0.033135005
LOC6429	-0.089615244	6.524182486	-3.10989614	0.002072886	0.0442738
UBE2O	-0.089590206	7.282253425	-3.102291705	0.002124968	0.044813576
PAFAH1E	-0.088930688	6.784138236	-3.454999508	0.000639329	0.022310752
SLC44A5	-0.087800969	6.597835292	-3.167880504	0.001712841	0.038960921
SPRY4	-0.087740715	6.857935751	-3.374373083	0.000848932	0.026073793

STRN	-0.08713395	7.196080407	-3.173101079	0.00168344	0.03869271
MUM1L1	-0.086835513	6.473716131	-3.420677595	0.000721821	0.023994813
PLCH1	-0.085838598	6.518096246	-3.103006524	0.002120022	0.044791572
LOC73027	-0.085432288	6.599844473	-3.404479107	0.000764115	0.024873935
RAG1	-0.085373767	6.569903144	-3.916226568	0.000114117	0.008011159
LOC40001	-0.084820154	7.896796386	-3.651955397	0.000312784	0.014908444
LOC10021	-0.084743362	6.525083417	-3.196066072	0.00155957	0.037006937
GABRR1	-0.083637463	6.52113568	-3.330084259	0.000989777	0.028420089
ROBO4	-0.083338511	6.619063556	-4.266568559	2.75E-05	0.003270274
MAPKAP	-0.083162922	8.362894953	-3.104191554	0.002111844	0.044710029
LOC65253	-0.083043932	6.60223008	-3.365715937	0.000874901	0.02658948
LOC73177	-0.082924649	7.508232264	-3.252616746	0.001289624	0.033197423
LOC10013	-0.081542978	6.582436243	-3.545479931	0.000462154	0.01839659
ST6GALN	-0.079877109	6.464382583	-3.402474092	0.000769508	0.02501244
SSB	-0.079718447	7.927063248	-3.315263043	0.001041576	0.029304147
PSMD11	-0.079692766	7.257590343	-3.049819893	0.002518538	0.04927858
LOC10013	-0.079106136	6.614258362	-3.2999888	0.001097588	0.030222181
BAALC	-0.078763632	6.529241413	-3.264070664	0.001240526	0.032450092
COL13A1	-0.077634024	6.698902724	-3.329593121	0.000991455	0.028420089
YIPF4	-0.077297763	7.663016393	-3.461497572	0.00062474	0.022105331
HERPUD2	-0.077215077	7.0983963	-4.076004865	6.04E-05	0.005371554
FBXO11	-0.07698488	7.882346067	-3.462940443	0.000621543	0.022088935
CYTH3	-0.076632964	7.088968914	-3.329739945	0.000990953	0.028420089
SCAND3	-0.076340448	6.525943943	-3.505397839	0.000534045	0.02005909
HK1	-0.075951057	8.857005472	-3.047119605	0.002540501	0.049444144
FAM126A	-0.075688055	6.716513562	-3.622724476	0.000348486	0.015940668
SPATA7	-0.073631032	6.995609271	-3.073031832	0.002336822	0.047280837
MXRA7	-0.071624095	6.945489625	-3.082415977	0.002266847	0.046451365
ATP6V1H	-0.07110271	7.356455539	-3.148476103	0.001826332	0.040613722
LYST	-0.071000105	6.940780371	-3.676332894	0.000285672	0.014233732
INPP5A	-0.070473683	7.102409615	-3.95803398	9.68E-05	0.007348516
LOC72881	-0.067391319	6.497230076	-3.43785378	0.000679369	0.023172955
DDX52	-0.065161776	7.609496165	-3.064376794	0.002403118	0.047829454
LOC64481	-0.065142501	6.694245235	-3.248072488	0.001309599	0.033499207
AADACL4	-0.063985146	6.527879183	-3.208089535	0.001498135	0.036214004
TBX1	-0.063915172	6.685020295	-3.389529396	0.000805192	0.025420229
DEFB126	-0.063106877	6.370582087	-3.339731179	0.000957362	0.028010804
LOC10013	-0.06276154	6.583126822	-3.409796342	0.000749983	0.024610007
GABPB1	-0.062597952	6.854591461	-3.092842315	0.002191367	0.045382575
SEC15L2	-0.060327486	6.633316466	-3.457863029	0.000632861	0.02220925
LOC65355	-0.059384137	6.891108522	-3.104134213	0.002112239	0.044710029
LOC72836	-0.059215867	6.605216814	-3.525856209	0.000496134	0.019210951
SLCO1A2	-0.058207881	6.442563994	-3.316244014	0.001038071	0.029242984
R3HDM2	-0.056790168	6.890308966	-3.524583853	0.000498417	0.019210951
RPGR	-0.055836924	6.850355754	-3.347768037	0.000931115	0.02748785
C3orf36	-0.055333228	6.640676976	-3.352508276	0.00091595	0.027325368
SNORD12	-0.054802654	6.596630807	-3.165237333	0.001727907	0.039141538
NAP1L2	-0.052232489	6.522002783	-3.380867485	0.000829925	0.02582995
C22orf9	-0.04788332	6.691144036	-3.347498983	0.000931982	0.02748785
NR2E3	-0.046709729	6.365947108	-3.524403203	0.000498741	0.019210951
SLC35E4	-0.042574383	6.470132198	-3.605228276	0.000371654	0.01641343

FKBP9	-0.041029669	6.493040997	-3.141836317	0.001866736	0.041265382
H2BFWT	-0.039755952	6.339129834	-3.379571431	0.000833686	0.025873713
LOC6449	-0.037333217	6.515127138	-3.147178325	0.001834165	0.040709208
LOC3398	-0.033801673	6.374714726	-3.056448159	0.002465365	0.048746681
TBRG1	-0.033537958	6.487555082	-3.454843346	0.000639684	0.022310752
LOC3997	-0.033137899	6.410102462	-3.620657165	0.000351151	0.015941807
LOC1001	-0.026088832	6.425393118	-3.095849767	0.00217003	0.045177126
ZMAT1	0.025459526	6.394749644	3.65718097	0.000306776	0.014653869
ASCL3	0.025505499	6.373765145	3.063590607	0.002409225	0.047907609
CDKN2C	0.028452942	6.460698896	3.070879952	0.002353146	0.047485658
WNK3	0.028496489	6.440189625	3.442787853	0.000667613	0.022956898
FLJ16734	0.030030174	6.386035813	3.504277888	0.000536197	0.020096525
CCDC155	0.030302532	6.394599216	3.509658764	0.000525932	0.019822138
MIR488	0.030935691	6.473905384	3.529075117	0.000490405	0.019105779
ZNF667	0.031063817	6.459754376	3.4911542	0.000562024	0.020548
GLYAT	0.032091625	6.425260513	4.055188803	6.57E-05	0.005652985
NUDT6	0.03418221	6.555363437	3.495166478	0.000554007	0.020393018
DDX25	0.034307023	6.377402565	3.452401168	0.000645252	0.022362964
MIR1979	0.034325172	6.510794949	3.169749498	0.001702261	0.03888127
NEURL	0.035750512	6.348648279	4.46175858	1.20E-05	0.002006922
TLX3	0.035757151	6.390848899	3.272742662	0.001204513	0.031849304
LOC7294	0.03593853	6.501744778	3.278312379	0.001181898	0.031631978
SENP1	0.035975875	6.492807205	3.273996308	0.001199388	0.031802072
IGSF5	0.037249713	6.346540753	3.215480968	0.00146149	0.035641853
N4BP3	0.037367686	6.381087119	3.389952584	0.000804001	0.025419163
PRR12	0.037698886	6.533777236	3.550931448	0.00045311	0.018156921
ZNF580	0.038534882	6.855136119	3.243881272	0.001328276	0.033819473
SMAD2	0.038578295	6.678520793	3.305002958	0.001078899	0.030036156
HESX1	0.038598861	6.392888802	3.252474117	0.001290247	0.033197423
GBX1	0.038714572	6.384550037	3.895153398	0.000123924	0.008492826
LOC6427	0.040879827	6.408279745	3.178008656	0.001656229	0.03832208
ZNF571	0.04115884	6.593205509	3.289897902	0.001136116	0.030743679
LOC7291	0.041434281	6.508038431	3.079444211	0.002288795	0.046782965
LOC2545	0.041815527	6.391813131	4.948159051	1.32E-06	0.000427626
CD8B	0.042252696	6.403117672	3.092176305	0.002196119	0.045438148
RIBC2	0.042446672	6.31316584	4.458759793	1.21E-05	0.002017829
LOC7288	0.042580685	6.439267721	3.204852633	0.00151445	0.036358362
BAT4	0.043247298	6.512816256	3.543726446	0.000465099	0.018446986
NFATC2I	0.043255204	7.039922836	3.317193946	0.001034687	0.029185086
CPEB1	0.044008918	6.378147072	4.397983647	1.58E-05	0.002371944
FLJ16793	0.044126592	6.480381367	3.262754307	0.001246079	0.032520148
UBL7	0.044233903	6.895990188	3.10198891	0.002127067	0.044813576
ZNF414	0.044298212	6.60157817	3.584673513	0.00040072	0.016998119
LOC7310	0.044448038	6.386090339	3.666271223	0.000296582	0.014442048
FLJ37453	0.044511231	6.437407562	4.440880437	1.31E-05	0.002086213
PCLO	0.04474392	6.448390471	3.154400407	0.001790966	0.040197041
GIPC2	0.045095954	6.399855344	3.570829188	0.000421487	0.017507242
PRSS36	0.045266942	6.532308119	3.980919453	8.84E-05	0.006953671
NCR3	0.045754523	6.336244272	3.650070554	0.000314978	0.014980528
GRK6	0.046377206	6.594377362	3.810843948	0.00017174	0.010367173
TTLL3	0.04657065	6.711691189	3.290605508	0.001133373	0.030740308

RAB37	0.047227239	6.510310516	3.218948808	0.001444584	0.035337772
PDE6B	0.047428388	6.440151976	3.293148195	0.001123571	0.030559002
ZNF519	0.047912425	6.503584663	3.168066582	0.001711785	0.038960921
GZMM	0.048691143	6.465115917	3.302109235	0.001089648	0.030141967
ICAM4	0.048799244	6.475798363	4.287119695	2.53E-05	0.003121601
KIF24	0.049014133	6.564773074	3.153723045	0.001794978	0.040204938
SMC1A	0.049097524	6.54652495	3.257699888	0.001267617	0.032807229
ONECUT2	0.049313032	6.473691914	3.26273419	0.001246164	0.032520148
GRAP2	0.049371965	6.36983173	3.6141436	0.000359673	0.016183947
PRELID2	0.049482185	6.388134285	3.791396398	0.000185018	0.01084104
DTNBP1	0.049948807	6.637547679	3.340508029	0.000954795	0.027972949
PRAM1	0.050587085	6.402752779	3.569444977	0.000423618	0.017562557
CIT	0.051280849	6.485547187	3.322964868	0.001014352	0.028850133
CTSE	0.051826931	6.444874587	3.291025417	0.001131749	0.030739089
PSTK	0.051939501	6.715476531	3.175584671	0.001669618	0.038536254
ZPBP2	0.05198452	6.359238364	5.766830799	2.22E-08	1.87E-05
LOC10012	0.05200109	6.500359908	3.322798047	0.001014934	0.028850133
DHFR	0.052959808	6.607587572	3.239699568	0.001347156	0.033976898
ECE1	0.053207602	6.53726174	3.334061509	0.000976291	0.028365183
LOC72959	0.053340313	6.591559459	3.526258249	0.000495415	0.019210951
LIPE	0.053542382	6.516947156	3.377619849	0.00083938	0.025977031
EFCAB7	0.053896295	6.71214466	3.048065188	0.002532791	0.049444144
CNTD1	0.054224097	6.373696327	4.646695408	5.29E-06	0.001203451
WDHD1	0.054308405	6.683614467	3.119552356	0.002008448	0.043181642
IKZF4	0.05432242	6.700956371	3.359455618	0.000894139	0.02698752
LCK	0.054376484	6.578581351	3.293107234	0.001123728	0.030559002
TBC1D17	0.05453717	6.583367342	3.238059261	0.00135463	0.033976898
CASP9	0.054539985	6.874014977	3.319243729	0.001027421	0.02912971
FANCC	0.054653009	6.550097206	3.202869035	0.001524529	0.036411388
RAB33A	0.05500716	6.492726588	3.166629224	0.001719958	0.039082349
MIER1	0.05535882	7.235297382	3.349193519	0.00092653	0.02748785
LOC64874	0.05550145	6.55962916	3.199721318	0.001540651	0.036695481
POGZ	0.055544632	6.731309085	3.220646115	0.001436376	0.035185616
S100PBP	0.055731547	7.251947257	3.290290866	0.001134592	0.030740308
TMEM129	0.055807767	6.699911009	3.251239062	0.00129565	0.033297449
ESCO2	0.056402638	6.493261942	3.157140051	0.001774827	0.040017302
ZFR2	0.056787105	6.433293813	4.629351854	5.72E-06	0.001268653
HMSD	0.056966745	6.387041159	3.748944763	0.000217446	0.012004893
HAUS6	0.05797838	6.585261667	3.238757357	0.001351445	0.033976898
TOE1	0.058097879	6.740705897	3.318667495	0.001029459	0.029149874
FLJ46380	0.058360533	6.545145101	3.181140689	0.001639076	0.038031053
USP4	0.058466945	6.985643886	3.437498239	0.000680224	0.023172955
SASS6	0.058882425	6.946266002	3.074983401	0.002322108	0.047112864
C10orf75	0.059282866	6.606391655	3.293304936	0.001122969	0.030559002
CNTLN	0.059416229	6.615305321	3.560816633	0.000437131	0.017863091
KIAA2022	0.059416915	6.400336523	4.205373057	3.55E-05	0.00388523
FLJ21687	0.059490905	6.604789974	3.043558252	0.002569737	0.049795306
SAMD3	0.059691709	6.485467835	3.294285891	0.00111921	0.030559002
KATNAL1	0.059871206	6.490893125	3.079569215	0.002287868	0.046782965
SLC25A35	0.060099094	6.504593875	3.398839643	0.000779375	0.025095509
LOC34027	0.060598663	6.537260073	3.452835156	0.000644259	0.022362964

PIK3R3	0.060981971	6.565444848	3.800091807	0.000178965	0.01073549
LOC64321	0.061706378	6.413827292	4.665991921	4.85E-06	0.001158319
ATG10	0.06206171	7.234260046	3.109512379	0.002075486	0.0442738
OGG1	0.062670355	6.569975853	5.456653392	1.10E-07	6.37E-05
MPP2	0.062840427	6.456945146	3.823152991	0.000163808	0.00997051
SAMD10	0.062961205	6.654704614	3.719514562	0.000243002	0.01277386
ZNF471	0.063280816	6.490013296	3.043867103	0.00256719	0.049795306
NFIC	0.06337431	7.071026067	3.065249695	0.002396354	0.047829454
LOC64391	0.063400635	6.614420457	3.184963027	0.001618365	0.037830134
ASRGL1	0.063419882	6.411812861	3.497867136	0.000548671	0.020364765
LY75	0.063523901	6.571267774	3.09756756	0.002157928	0.045115283
DTNB	0.063803497	6.599894335	3.063221426	0.002412098	0.047921365
C19orf57	0.064441655	6.436634368	6.82072962	5.98E-11	7.30E-08
RNF207	0.064853898	6.639321117	3.332733746	0.000980774	0.028399438
CAMK1	0.065451907	6.674485742	3.386256974	0.000814454	0.025545532
ANKRD4	0.065478306	6.476805418	3.113861377	0.002046198	0.043936433
E2F8	0.066046076	6.498110635	3.952806866	9.88E-05	0.007461574
ZFP14	0.06606136	6.587788281	3.101384651	0.002131261	0.044813576
LOC1001	0.066457706	6.623042281	3.248592855	0.001307297	0.033479303
SGOL1	0.066596548	6.749712044	3.10833469	0.002083483	0.044317888
LDHC	0.066615596	6.53917266	3.232087506	0.001382165	0.034433472
TCERG1L	0.066981636	6.377388506	3.108990226	0.002079028	0.0442738
FASLG	0.067164579	6.457687637	3.770785879	0.000200145	0.01139321
ATP6AP1	0.067176718	6.653429116	3.269263383	0.001218844	0.032073834
TXNDC1	0.067684212	6.556181011	4.253368473	2.91E-05	0.003419267
LOC6444	0.068551718	6.688726834	3.662603803	0.000300656	0.014442048
PARP16	0.069219578	6.79563621	3.352327184	0.000916525	0.027325368
LOC6434	0.069647631	6.548734898	4.036443379	7.08E-05	0.00602272
C20orf96	0.069860199	6.552990834	4.162223517	4.25E-05	0.004342387
ZNF473	0.070271788	7.154917863	3.381919613	0.000826883	0.025771788
CD1E	0.071420941	6.612618634	3.13233781	0.001925972	0.042108836
PSMC3IP	0.072112515	6.812481375	3.134662547	0.001911316	0.041913513
IL16	0.072470178	6.484880306	3.127209678	0.00195867	0.042527515
GKAP1	0.072544577	6.553614965	3.888440824	0.000127212	0.008550166
TMCO4	0.072678209	6.711199164	3.68056897	0.000281193	0.014135531
WDR76	0.072851373	6.558957788	4.107239818	5.32E-05	0.004952608
HPDL	0.072994503	6.517675209	3.129566064	0.001943582	0.042357967
VRK3	0.073182258	7.065307714	3.400018427	0.000776162	0.025080311
SLC1A1	0.074253798	6.478391136	3.669121298	0.000293453	0.014386681
TOPORS	0.074283465	7.119210233	3.211804976	0.00147961	0.035845051
MAMDC4	0.074569889	6.517523501	3.962578029	9.51E-05	0.007278882
RCADH5	0.074890115	7.006081405	3.076143654	0.0023134	0.046979988
LOC6455	0.074899269	6.523418904	3.867491975	0.000138014	0.008902581
SLC16A8	0.074989072	6.382863094	4.322782658	2.17E-05	0.002858244
LOC2837	0.075686957	6.987303851	3.930364069	0.000107955	0.007777378
ZNF488	0.075690543	6.525511135	3.077023746	0.002306816	0.046889602
UBASH3	0.075874693	6.428839343	3.797520083	0.000180736	0.010762336
LOC1001	0.076094107	6.456815344	5.540304195	7.20E-08	4.52E-05
ZNF300	0.076216332	6.551251262	3.15535717	0.001785315	0.040152217
TRIM74	0.076270753	6.47162541	5.252303343	3.06E-07	0.000149365
SYNJ1	0.076493679	7.209272906	3.950475284	9.97E-05	0.007489916

VILL	0.076618492	6.511482057	3.758956098	0.000209347	0.011691789
ZNF684	0.076638983	6.833222984	3.196269633	0.00155851	0.037006937
FIZ1	0.076655239	7.36310636	3.195230345	0.001563925	0.037070261
C14orf28	0.077465203	6.880389506	3.056119001	0.002467981	0.048746681
ATXN7L2	0.077803453	6.908580624	3.333786086	0.000977219	0.028365183
UBXN11	0.077841914	6.759354329	3.914021949	0.000115107	0.008054954
PHF17	0.078829654	6.842241909	4.565735098	7.58E-06	0.001501482
VWCE	0.078900682	6.518403744	3.139354978	0.001882046	0.04147863
SLC25A29	0.079104618	6.861515825	3.123496782	0.001982662	0.042878978
KIAA1328	0.07940948	6.621412555	4.842424337	2.17E-06	0.000643702
C19orf36	0.080097406	6.521345549	3.346575311	0.000934967	0.027538916
WDR7	0.080113748	7.099909882	3.621800351	0.000349675	0.015940668
INPP5B	0.080153552	6.890556891	3.939049237	0.000104328	0.007621426
LOC28636	0.080675531	6.638439872	3.329042389	0.000993339	0.028420089
PHF1	0.080721098	7.001685423	3.7463122	0.000219625	0.012086891
C7orf13	0.081294798	6.622249746	3.6281287	0.000341609	0.015835804
IRF2	0.081471739	7.102704628	3.391381095	0.000799994	0.025412709
PDCD1	0.081887915	6.506669211	3.08221245	0.002268344	0.046451365
KIAA1641	0.082314849	6.885357336	3.426232863	0.000707827	0.023817889
LOC40101	0.082799763	12.68253217	3.585503356	0.000399506	0.016998119
RFC1	0.083284351	7.658358263	3.458922268	0.000630484	0.02220925
PARD6A	0.083296799	6.526618344	4.645695628	5.31E-06	0.001203451
SAMD1	0.083490029	6.863031594	3.546987589	0.000459636	0.018362882
LOC10012	0.083523937	6.582711091	4.30974572	2.30E-05	0.002984673
ATPAF1	0.08355672	7.841794046	3.334954326	0.000973287	0.028325884
MAT2B	0.08384672	7.894996432	3.064673549	0.002400817	0.047829454
FAM159A	0.08387541	6.408785057	4.060946467	6.42E-05	0.005573257
HLCS	0.084411396	6.74879316	4.223217906	3.30E-05	0.00371425
PWWP2A	0.085197755	7.253444422	3.394853979	0.000790331	0.02527794
MYO5B	0.085356414	6.801381584	3.132407466	0.001925531	0.042108836
CHADL	0.085460539	6.607380217	4.465940951	1.17E-05	0.001985932
MORC4	0.085619514	6.952203793	3.241752082	0.001337858	0.033976898
SIRPG	0.085774273	6.478235857	3.672914995	0.000289335	0.014283147
ZNF540	0.085965734	6.449165755	4.227913177	3.24E-05	0.003684429
WHSC2	0.08642459	6.998615851	4.120991472	5.03E-05	0.004804771
ALDH16A	0.086495326	7.351310104	3.310256166	0.001059636	0.029698203
CNOT10	0.086862472	7.339312149	4.020748861	7.54E-05	0.006301382
ANKRD34	0.086923516	6.34396531	4.675443036	4.65E-06	0.00113168
HMX2	0.087367683	6.48703152	4.330339802	2.10E-05	0.002808838
TRIM45	0.08749749	6.714906837	3.928513151	0.000108744	0.007808572
RAB28	0.088109012	7.004257986	3.698270134	0.000263182	0.013448603
GOLT1A	0.08811893	6.487490659	3.996645642	8.30E-05	0.006683973
ELF2	0.088593574	7.339518147	4.088215432	5.75E-05	0.005175237
SNRNP25	0.088608731	6.953543859	3.570936627	0.000421322	0.017507242
DCLRE1B	0.089041774	6.628667807	4.882302514	1.80E-06	0.000557487
PRRT3	0.089509152	6.724686868	3.387592056	0.000810663	0.0254881
LOC65212	0.089555242	6.46271971	3.538704405	0.000473632	0.018650745
GPT	0.089748942	6.497529452	3.250167625	0.001300354	0.033379305
RNPC3	0.090082197	7.339597988	3.597421762	0.000382451	0.016510025
C21orf58	0.090474801	7.439200497	3.54100946	0.000469698	0.018574851
YPEL1	0.090477909	6.557507178	4.296872143	2.42E-05	0.003048284

SP5	0.090890472	6.425097332	3.52377132	0.000499879	0.019210951
RBBP4	0.090902224	7.566624983	3.969795901	9.24E-05	0.007127413
LOC64428	0.091068378	6.600157206	4.383402158	1.68E-05	0.002474228
APOC2	0.091127914	6.556408551	3.774775331	0.000197128	0.011257239
LOC6523?	0.091196462	6.4286615	5.676109334	3.57E-08	2.66E-05
SIGIRR	0.091276605	6.776882695	5.181728441	4.32E-07	0.000193192
FBXW5	0.091496712	7.102658749	3.726982987	0.000236261	0.012569873
BUB3	0.091563027	8.631053574	3.176427378	0.001664952	0.038468965
MUTED	0.09220201	7.858131277	3.094568614	0.002179096	0.045177126
MRPS36	0.092600603	6.850918588	3.69409452	0.00026733	0.013597319
PMF1	0.092603296	7.164232629	3.88188038	0.000130506	0.008689698
BAX	0.0928245	7.25291656	3.598545358	0.000380879	0.016474527
LOC9249?	0.09294031	6.652821155	4.730996789	3.61E-06	0.000945495
VHL	0.09304332	7.370013587	3.851960981	0.000146577	0.00917989
DBP	0.093287053	6.799119475	3.861030903	0.000141517	0.008975489
FANCD2	0.093420495	6.970377558	4.394321	1.60E-05	0.002393156
LOC65128	0.09358808	6.544789355	3.839493316	0.000153812	0.009574269
PYHIN1	0.094342422	6.647542403	3.232735268	0.001379154	0.034397442
CABLES2	0.094402115	6.943838325	3.151984389	0.001805312	0.040310997
TAPT1	0.094483051	7.366971899	3.153947509	0.001793647	0.040204938
FAM111B	0.094501251	6.581973245	4.50729095	9.81E-06	0.001766816
TADA3	0.094519582	7.599632337	4.054500393	6.59E-05	0.005652985
USP37	0.094569874	7.540499489	3.413300589	0.000740804	0.024378479
MGA	0.094801108	6.957948933	5.646431362	4.16E-08	2.87E-05
TSPAN32	0.095606471	6.618469098	3.474787117	0.000595867	0.021358866
KIAA171?	0.096004918	7.462445648	3.576018975	0.000413586	0.017408967
SLC4A2	0.096548476	7.322404213	3.064512126	0.002402068	0.047829454
STMN1	0.096673081	7.143634207	3.86547244	0.0001391	0.008902581
GPX1	0.097267907	8.253720339	3.365768565	0.000874741	0.02658948
MED12	0.097356826	7.620550787	3.350380203	0.000922729	0.027473069
NOXO1	0.097638918	6.691214298	3.20735001	0.001501848	0.036224055
APOBEC3	0.097666558	6.541419863	3.534354785	0.000481141	0.018822333
RNF5	0.098102095	7.170250638	4.115682255	5.14E-05	0.004853234
C21orf45	0.098803804	7.271534342	3.422712229	0.000716666	0.023932065
PCBD2	0.100014381	6.870537814	3.788111853	0.000187354	0.010919681
GPAT2	0.100036777	6.416522288	5.947767782	8.44E-09	8.06E-06
BCOR	0.100214258	7.263519971	3.672378568	0.000289914	0.014283147
HNRPH3	0.100680982	7.706336966	3.407207966	0.000756832	0.024710055
UBE2D2	0.100750299	7.359506266	3.802725916	0.000177169	0.010665587
GIN1	0.100830009	6.796804826	3.982591167	8.78E-05	0.006941247
ALKBH7	0.101507768	7.22981037	3.400888177	0.000773799	0.025040788
ZNF33B	0.101722766	7.504673787	3.067917272	0.002375792	0.047805203
CLGN	0.102002022	6.395315117	4.374238007	1.74E-05	0.002539147
C5orf34	0.102285911	6.710452551	4.346419287	1.96E-05	0.002749869
MLH1	0.102327362	7.982659529	3.60187191	0.00037626	0.016416306
UBR7	0.102814677	8.095681264	3.498566297	0.000547297	0.020348157
LOC65318	0.103077872	6.429081161	5.595415707	5.42E-08	3.54E-05
RASSF1	0.103134676	7.072638523	4.334178458	2.07E-05	0.002790174
DMXL1	0.103353332	6.887584934	3.87032528	0.000136504	0.008902581
FAM98C	0.103395559	7.113779936	3.932611977	0.000107005	0.007734283
CNNM3	0.10352206	7.426927199	3.173892608	0.001679024	0.038631606

FLJ13305	0.103622414	6.769655245	3.279607189	0.001176698	0.031567639
B9D2	0.103840747	7.511188554	3.095158132	0.00217492	0.045177126
ZNF232	0.104276605	7.180926641	3.389976904	0.000803933	0.025419163
ABHD14E	0.104569513	7.044480564	3.088063017	0.002225674	0.045833861
ZNF248	0.104899255	7.163770936	3.374992376	0.000847102	0.026069161
GUSBL1	0.105037479	7.630214862	3.090236435	0.002210012	0.045596796
PHF19	0.105149316	7.284206108	3.872104081	0.000135564	0.008891765
UBE3A	0.106121808	8.991709344	3.258119723	0.001265814	0.032799224
RTKN2	0.106492912	6.566220908	4.804258155	2.59E-06	0.000723965
C18orf32	0.10667965	7.707658244	3.46334736	0.000620644	0.022088935
EPM2AIP	0.106839518	7.762098829	3.061672664	0.002424184	0.048117968
CETN3	0.106919491	7.365555654	3.331475412	0.00098504	0.028420089
PPM1D	0.107098275	7.545342598	3.120426897	0.002002705	0.043142579
C9orf116	0.107105158	6.671452289	3.329052449	0.000993304	0.028420089
LOC6468(	0.107120588	6.964677633	3.585064747	0.000400148	0.016998119
C19orf62	0.107146636	8.048834829	3.573877739	0.000416829	0.017412518
RBBP9	0.107264132	7.292416699	4.233583637	3.16E-05	0.003636067
AKR7A3	0.107389218	8.286842512	3.183835735	0.001624447	0.037872573
SMPDL3E	0.10751668	6.538522302	4.341973201	2.00E-05	0.002749869
MTMR14	0.107563492	7.592255739	4.115759222	5.14E-05	0.004853234
ZBTB5	0.107891973	7.66078344	3.129331705	0.001945078	0.042357967
CRYGS	0.107984656	6.990087029	3.483820316	0.00057696	0.020811935
RNF5P1	0.108427426	7.553094252	3.923126584	0.000111069	0.00787265
WDR51B	0.10848412	7.569200117	3.500414629	0.000543682	0.020316862
C14orf73	0.109029076	6.615557114	3.174465071	0.001675836	0.038631606
TBCD	0.109124525	7.425070861	3.154521917	0.001790248	0.040197041
DUT	0.109272679	7.395512712	3.626764666	0.000343332	0.015848832
ZWINT	0.109559066	7.26158078	3.621168275	0.00035049	0.015941807
PYGO2	0.109696574	7.063124178	4.324216916	2.16E-05	0.002858034
APITD1	0.110136132	7.335827244	3.498932617	0.000546579	0.020348157
FAM178A	0.110189226	7.784602773	3.064459677	0.002402475	0.047829454
HMGN2	0.110756789	8.178152047	3.977701764	8.95E-05	0.007001783
BRD8	0.110948383	8.071861375	3.360157561	0.000891962	0.026958857
AMY2B	0.110960631	6.771560815	3.417681833	0.000729474	0.024160782
FAF1	0.111092549	8.019782428	3.642469453	0.000323974	0.015308982
C19orf25	0.111182553	7.120165232	3.823921865	0.000163324	0.00997051
APPL1	0.111186571	7.704867112	3.288661064	0.001140923	0.030835807
AGL	0.111192534	7.737036672	3.721052437	0.000241599	0.012761182
ZNF564	0.111265833	7.271085828	3.678421153	0.000283456	0.014187639
LOC6542(	0.111278534	7.06587579	3.789702016	0.000186219	0.010882429
XRCC1	0.11146259	7.279059264	3.627007	0.000343026	0.015848832
C6orf190	0.111810226	6.601306669	3.168307883	0.001710416	0.038960921
FAM53B	0.112081023	7.421673564	4.499558049	1.01E-05	0.001798149
C19orf43	0.112086866	9.425970069	3.495133341	0.000554072	0.020393018
NUP62	0.112258451	8.802246455	3.142227621	0.001864332	0.041253746
LOC1001:	0.112683197	7.645692273	5.110648003	6.10E-07	0.000235199
HMHA1	0.11269011	6.989584326	3.1231454	0.001984947	0.042886177
CREB3L4	0.113250291	6.660257496	4.385669851	1.66E-05	0.002466856
SUOX	0.113299858	7.740018918	3.044859303	0.002559021	0.049722918
TRAF2	0.113350215	6.908558509	3.317405073	0.001033937	0.029185086
RMND5B	0.113378579	7.281829041	3.730557366	0.000233097	0.012492315

DMRTA2	0.113403789	6.549483346	3.744163666	0.000221418	0.012132691
CENPM	0.11475773	7.06179582	3.798881881	0.000179796	0.01073549
GPR18	0.114798458	6.497288041	3.370982591	0.000859016	0.026288513
SLC6A16	0.115113616	6.728929568	3.423945137	0.000713559	0.023900958
SP1	0.115465976	8.378717376	4.01489341	7.72E-05	0.0063791
ANKRD3(	0.115746063	7.116795207	3.688289919	0.000273199	0.013863725
TATDN2	0.115941605	7.875086489	3.617218156	0.000355626	0.016111709
EXOSC9	0.116644263	8.37749801	3.356071882	0.000904701	0.02720647
KLF11	0.116839278	7.393912156	3.69461842	0.000266806	0.013597319
HNRNPAI	0.116950271	9.490829017	3.09141971	0.002201528	0.045507212
ASTE1	0.11708445	7.369622103	3.212719126	0.001475084	0.035845051
GLS2	0.117219412	6.513053511	5.72336428	2.78E-08	2.27E-05
LGALS9	0.117778607	6.706667026	4.025429142	7.40E-05	0.006207969
GPBP1	0.117879174	8.673893126	3.23774128	0.001356083	0.033976898
LOC4409:	0.117898942	9.98169886	3.446204418	0.000659584	0.022716365
SLC27A5	0.11802934	6.784346472	3.301131361	0.001093303	0.030141967
PIN1	0.118059385	9.524595077	3.458576176	0.00063126	0.02220925
TCF19	0.118063891	6.720907763	5.216940468	3.64E-07	0.000166592
SLC25A3:	0.11885865	6.96692361	3.343085656	0.000946324	0.027822328
CXCR3	0.119114764	6.716972405	3.335347751	0.000971966	0.028324959
FLJ25996	0.119641461	6.591527286	3.413175767	0.000741129	0.024378479
SSX2IP	0.120059969	6.762568581	4.455741661	1.23E-05	0.002029068
NFYC	0.120065461	8.329957042	3.662441188	0.000300838	0.014442048
FAM116A	0.120819906	8.483076859	3.601211135	0.000377174	0.016416306
MDK	0.120943568	7.021551469	4.223872454	3.29E-05	0.00371425
LRRC56	0.121001675	6.902906172	3.139788981	0.00187936	0.04147863
SYNGR3	0.121127717	6.392984293	8.01019199	3.50E-14	7.68E-11
TEX9	0.121233972	6.922797689	3.205473617	0.001511308	0.036358362
RAPGEF6	0.121354888	7.848793538	3.280336874	0.001173777	0.031529819
ENPP6	0.121677431	6.45424412	3.413213432	0.000741031	0.024378479
LOC7296:	0.121722781	7.18957242	3.957541888	9.70E-05	0.007348516
LRDD	0.12219933	7.004217775	3.767936676	0.000202326	0.011428533
RSBN1	0.122891463	8.150131619	3.101919386	0.002127549	0.044813576
ZNF700	0.123206502	8.012681371	3.228456247	0.001399162	0.034748138
LOC6449:	0.123857924	8.053574992	4.094269313	5.61E-05	0.005070412
WRB	0.124082692	7.746465602	3.185924968	0.001613191	0.037749351
ZNF524	0.124761562	7.418266445	3.866231891	0.000138691	0.008902581
CRBN	0.124860956	7.725595425	3.273185305	0.001202701	0.03183971
METTL4	0.125339844	7.133948998	3.940578071	0.000103701	0.007621426
C9orf82	0.126604073	7.21616504	4.280106834	2.60E-05	0.003175328
PTAR1	0.126900224	7.855662912	3.432554866	0.00069221	0.023473555
ZNF644	0.126958365	7.788555801	4.329735254	2.11E-05	0.002808838
GUSB	0.127175496	8.500782318	3.094866973	0.002176981	0.045177126
GRIN2C	0.127672259	6.47635614	7.375157419	2.04E-12	2.99E-09
CST7	0.127916115	7.056222999	3.065833836	0.002391838	0.047829454
POLA1	0.128135434	7.864648301	3.224623038	0.001417314	0.034913263
MRPS27	0.128210919	8.691821135	3.566459841	0.000428248	0.017643328
ZNF260	0.12893975	7.314708444	3.682593128	0.000279076	0.014064539
HNRPUL1	0.130231765	9.784066856	3.304410551	0.001081091	0.030036156
NR1D2	0.130372879	7.273317648	3.483892014	0.000576812	0.020811935
RAB33B	0.130935122	7.729405596	3.987902894	8.60E-05	0.006838213

NXT2	0.131018599	8.110862799	3.103860298	0.002114127	0.044710029
ANKRD3(	0.1311058	6.869346033	3.769012499	0.000201499	0.011411205
AMY2A	0.131611354	6.796752868	4.133010105	4.79E-05	0.004622417
CHCHD4	0.131639206	8.42553807	3.611417395	0.000363298	0.016192165
DONSON	0.131656433	7.296987747	3.523069228	0.000501146	0.019210951
FAM81A	0.131834866	6.475012219	4.246910281	2.99E-05	0.003494191
CLK4	0.132009278	7.365712386	3.259799556	0.001258628	0.032651514
FRG1	0.132291013	9.465405809	3.047336379	0.002538732	0.049444144
SMARCC	0.132949508	9.530588859	3.269351167	0.00121848	0.032073834
LCOR	0.1330065	8.815534499	3.239469625	0.001348202	0.033976898
LOC2017	0.133265657	7.297109853	3.720147421	0.000242423	0.01277386
ZFYVE20	0.133462662	8.586410188	4.27565137	2.65E-05	0.003199806
PPA2	0.133561488	10.17944332	4.219255533	3.35E-05	0.003729274
CCNE2	0.13360552	7.107253092	3.629420881	0.000339984	0.015793781
ATG7	0.133612886	8.275894503	3.211982151	0.001478731	0.035845051
LOC4414	0.133690517	7.436585334	3.712585651	0.000249417	0.012996865
BRPF1	0.134000577	8.286349156	5.241164119	3.23E-07	0.00015435
SS18L2	0.134784471	10.51352898	3.496276039	0.000551808	0.020393018
HIRIP3	0.135209457	7.821667341	3.778040553	0.000194692	0.01119884
ANKRA2	0.135420557	8.019092762	3.348697067	0.000928124	0.02748785
PRMT2	0.135690839	7.650220594	3.933728205	0.000106536	0.007725808
C19orf46	0.135982225	6.684511412	3.202918759	0.001524276	0.036411388
LOC1001	0.136017319	9.120750989	3.366777936	0.000871676	0.026564952
SYCE2	0.136571981	6.451129564	7.916248948	6.46E-14	1.18E-10
PCNA	0.136600087	8.297488249	3.740936337	0.000224137	0.012181382
HNRNPH	0.136885696	8.398834496	3.058791218	0.002446818	0.048479641
PTN	0.137191917	6.784459552	3.229103782	0.001396117	0.034741649
NAP1L4	0.137222101	9.990563219	3.13955798	0.001880789	0.04147863
ZBTB8A	0.137958369	7.57844102	3.554998813	0.00044647	0.018100148
RNF32	0.13814446	6.610787765	5.810598322	1.76E-08	1.61E-05
LOC7309	0.138218705	7.090705841	3.414528978	0.000737611	0.024372211
SUMO3	0.138615657	10.82257328	3.109156775	0.002077898	0.0442738
ACTR1B	0.139328527	8.030096322	4.448906029	1.27E-05	0.002059161
XK	0.139541417	6.652140057	3.525655742	0.000496493	0.019210951
TMEM80	0.139936944	7.348268392	4.574011809	7.31E-06	0.00147403
FLT3LG	0.140255851	6.957848565	3.589139192	0.000394229	0.016885772
LOC7317	0.140459182	6.570428861	4.319256321	2.21E-05	0.002883999
MGC1576	0.140593585	6.943869699	5.150129431	5.04E-07	0.000207078
ISOC2	0.140702785	8.048000068	3.135755649	0.00190446	0.041818053
UQCRC1	0.140973352	9.213966199	3.580064591	0.000407524	0.017220261
TUSC4	0.141004981	8.400192691	3.293568312	0.001121959	0.030559002
LOC7295	0.141165898	7.505075686	4.075010539	6.06E-05	0.005371554
LOC5414	0.141691649	6.532838268	6.392854484	7.17E-10	7.50E-07
C5orf5	0.141759536	7.823809634	3.630929719	0.000338095	0.015739323
APOBEC	0.142182877	7.349017913	4.180153307	3.94E-05	0.004195649
MNS1	0.142384711	7.097214324	3.152887349	0.001799938	0.040234018
C3orf70	0.142716313	6.856954853	3.124936538	0.001973327	0.042719119
C9orf40	0.143169567	7.060583563	3.96117205	9.56E-05	0.007294074
LOC3928	0.143272654	6.768672326	3.382931232	0.000823969	0.025717423
NKX2-3	0.143319061	6.463163174	5.474073958	1.01E-07	5.99E-05
RPAIN	0.143660512	9.056287486	3.274283327	0.001198218	0.031802072

KIF15	0.143882911	7.393690753	3.560665959	0.000437371	0.017863091
UPF3B	0.144238077	8.12359988	3.520422194	0.00050595	0.019233981
ABCA2	0.145051167	6.702141545	4.674272516	4.67E-06	0.00113168
P4HTM	0.145369194	7.268227558	3.425014603	0.000710874	0.023847378
IL2RG	0.14573871	6.743925484	3.639869822	0.000327106	0.015423805
GINS3	0.145792384	8.016153771	3.237066174	0.001359174	0.034014945
HACL1	0.146132273	8.271685734	3.271505562	0.001209591	0.031906764
ACBD7	0.146487562	6.548741782	3.87803327	0.000132474	0.008741317
ZNF641	0.147116799	7.301966151	4.362150772	1.84E-05	0.002652281
NDUFA2	0.147241632	9.58739662	3.353141881	0.00091394	0.027325368
NR2C2AP	0.147485665	7.842073236	3.587335576	0.000396839	0.016964478
RAB26	0.147618351	6.778550769	3.699728203	0.000261748	0.013447362
CUL9	0.147761981	7.567945009	3.35894381	0.000895729	0.026998428
LOC34737	0.148023029	9.220396095	3.324601415	0.001008653	0.028783289
GPC2	0.148300129	6.985949447	4.109698655	5.27E-05	0.004924133
C3orf19	0.148384361	8.125883399	4.254256927	2.90E-05	0.003419267
APOBEC3	0.148477642	7.133623348	3.043541493	0.002569876	0.049795306
RPS6KA5	0.14854733	7.543044298	3.321490155	0.001019513	0.028942831
ATP5O	0.148784576	10.63475268	3.099955253	0.002141211	0.044907949
LIN9	0.149308467	7.299899006	3.866002286	0.000138814	0.008902581
C18orf22	0.149389236	7.120878295	5.057441296	7.88E-07	0.000279214
USP1	0.149899954	7.647937647	5.223744488	3.52E-07	0.000164556
JAK2	0.150106333	7.220454276	3.088447569	0.002222895	0.045819584
SRGAP3	0.150326388	7.081026849	3.49721557	0.000549954	0.020377961
GOT1	0.150856989	9.4309146	3.14496218	0.001847613	0.040966299
DCI	0.150868451	7.988517305	3.592905751	0.000388832	0.016719757
C18orf54	0.151025565	6.982727757	4.862461794	1.98E-06	0.000602889
LOC64882	0.151083406	7.418557459	4.13665127	4.72E-05	0.004606981
TSEN34	0.152240309	9.427412493	4.172775914	4.07E-05	0.004215145
GPR137C	0.152490639	6.546043419	6.545542864	2.99E-10	3.46E-07
SFRS10	0.152588857	9.865613709	3.980336338	8.86E-05	0.006953671
CHDH	0.152639229	6.689307113	4.15414999	4.39E-05	0.004445734
CTSW	0.15313462	6.815415077	3.924925766	0.000110287	0.007867986
SREBF1	0.153422435	7.781639994	3.132890413	0.001922479	0.042108836
DNAJC8	0.153654523	10.31843088	3.193471531	0.001573129	0.037168131
CD4	0.155451687	6.75950614	3.718553976	0.000243882	0.012789525
SETMAR	0.155854022	7.792463897	3.498930411	0.000546583	0.020348157
E2F7	0.155876008	7.403099621	3.065104547	0.002397478	0.047829454
C3orf10	0.155965887	8.582180725	3.129910373	0.001941386	0.042357967
PDIK1L	0.156086386	7.427429884	3.860621191	0.000141742	0.008975489
C19orf48	0.156164673	8.154081029	3.551085311	0.000452857	0.018156921
EBP	0.15623231	8.061317272	3.206368027	0.001506792	0.036303439
MYH14	0.156606501	6.990171385	3.30436413	0.001081263	0.030036156
C3orf14	0.156983788	7.228376886	3.052318984	0.002498368	0.049102534
RFX7	0.157488689	8.861077336	3.267268439	0.001227132	0.032176328
MYO3A	0.157667631	6.522567279	4.673589009	4.69E-06	0.00113168
C8G	0.158064452	6.565897936	4.214351674	3.42E-05	0.003768943
LOC10012	0.159776534	6.623634757	4.575939109	7.25E-06	0.00147403
HMGN1	0.160542014	10.62643068	3.451288773	0.000647803	0.022416025
ACADM	0.161279727	8.840643974	3.125860095	0.001967359	0.042650824
SMARCA	0.162054689	8.153273609	3.887584938	0.000127637	0.008550166

SH2D4A	0.162155029	7.178564266	3.613170637	0.000360963	0.016186601
HDHD2	0.162580779	8.269548096	3.757630916	0.000210403	0.011704257
CXCR6	0.163221173	6.691481151	3.888958997	0.000126955	0.008550166
ITGB3BP	0.163409818	7.43343375	4.104065922	5.39E-05	0.004995827
RBM17	0.163520701	8.519382844	3.285627167	0.001152797	0.031080257
DDB2	0.163747947	7.180240832	5.178262291	4.40E-07	0.000193192
PCDHB9	0.165010674	8.27740881	3.194298365	0.001568796	0.037145641
CAPG	0.165748684	8.110582687	3.347954626	0.000930513	0.02748785
LOC4403	0.16576368	6.683571187	3.296381798	0.001111218	0.030469592
SUV39H1	0.166168283	8.141078542	3.45747181	0.000633741	0.02220925
SFRS7	0.166559621	8.502505358	3.233716832	0.001374602	0.03434593
PCNT	0.166566663	8.674630268	3.533704577	0.000482272	0.018822333
ANKRD3	0.166614513	7.122306486	4.165205571	4.20E-05	0.004328244
DCP2	0.167072465	8.594809704	3.456069958	0.000636904	0.022284551
BCAT2	0.167923499	8.115274088	3.903331897	0.000120027	0.008319704
RFC5	0.167983504	8.54100126	3.216618664	0.001455923	0.035545559
TDRD10	0.168078744	6.527040714	5.672695546	3.63E-08	2.66E-05
KEL	0.168596909	6.577505467	3.121432181	0.001996121	0.043085234
LOC2840	0.168801596	7.697428862	3.204465854	0.001516411	0.036358362
STIL	0.168855485	8.417011735	3.156995751	0.001775673	0.040017302
POLR2I	0.169086863	8.917714451	4.012044524	7.81E-05	0.0063791
FAM164A	0.169110811	7.473341484	3.574429168	0.000415992	0.01741064
XPC	0.16925671	8.278236653	3.614014289	0.000359844	0.016183947
CD3G	0.169412258	6.869461921	3.728317258	0.000235075	0.012537154
LOC1001	0.169505502	10.0664608	3.813800166	0.000169803	0.010278449
LOC7280	0.170140615	9.601537459	3.409635381	0.000750408	0.024610007
RBBP7	0.171267261	9.200378845	3.473448169	0.000598718	0.021426122
SLBP	0.171365478	8.845023374	3.668639296	0.00029398	0.014386681
AES	0.171506718	8.587928326	4.047059753	6.79E-05	0.005802258
FLJ12078	0.172283281	7.666542657	3.60472075	0.000372347	0.01641343
APOBEC3	0.172976891	7.00980078	3.434629858	0.000687155	0.023372841
CXXC1	0.172986403	9.679621274	3.36688399	0.000871354	0.026564952
C4orf19	0.173088248	6.681605981	3.906180229	0.000118697	0.008253561
IER3IP1	0.173106503	8.628199033	3.893060692	0.00012494	0.008499431
LOC6516	0.173671598	9.432054113	3.559213586	0.000439686	0.017924332
CD3E	0.173699344	6.68886674	4.440818816	1.31E-05	0.002086213
WDR48	0.173763828	7.797507157	4.826682061	2.33E-06	0.000681397
LTA	0.173977462	7.039527832	3.625476374	0.000344968	0.01589094
ARHGEF1	0.174836328	7.860714881	3.25334536	0.001286448	0.033177379
POLD1	0.17518627	7.452382548	4.28576607	2.54E-05	0.003121601
ERI1	0.175410831	7.734341341	4.612738018	6.16E-06	0.001319697
PAPSS1	0.176157298	8.627783619	3.741731662	0.000223464	0.012181382
ZDHHC2	0.176503793	7.279085922	3.674375508	0.000287765	0.014268537
H2AFZ	0.17697177	11.57455438	3.487471549	0.000569478	0.020730252
EZH2	0.177237219	7.439698221	4.13072764	4.83E-05	0.004637562
IL17RB	0.177307405	6.590611117	5.410251244	1.39E-07	7.85E-05
FAM83E	0.178746197	6.900254521	3.184504529	0.001620836	0.037847643
LOC6479	0.179305523	6.664398973	4.814427694	2.47E-06	0.000704018
LOC6449	0.179322253	9.951894285	4.359833632	1.86E-05	0.002652281
SNX26	0.179589517	7.840200156	3.044819166	0.002559351	0.049722918
POLE2	0.180170669	7.860567686	3.483428811	0.000577767	0.020811935

LOC7288	0.180294146	10.60575118	3.165271053	0.001727714	0.039141538
HIGD2A	0.18168748	9.75167348	3.864214988	0.00013978	0.008902581
MAP3K1	0.182519365	9.668516044	3.272198336	0.001206745	0.031869959
MXD3	0.183335352	6.882428999	5.645400663	4.18E-08	2.87E-05
TP53	0.183382219	6.994935007	4.513304879	9.55E-06	0.001740999
FAM13B	0.183486742	8.379270574	3.703807422	0.000257775	0.013295985
ARHGEF	0.183646776	9.186689291	3.533755517	0.000482184	0.018822333
SH2D1A	0.184277875	7.336880333	3.099482381	0.002144512	0.044907949
CCDC106	0.18497672	7.84599153	3.221226766	0.001433578	0.035185616
OSTalpha	0.185015222	6.769504396	3.61408567	0.00035975	0.016183947
C11orf85	0.185351944	6.424321709	9.410595392	2.31E-18	1.27E-14
AKR1A1	0.185917419	8.72444573	5.34888906	1.89E-07	0.000101555
PAQR8	0.186597456	7.234864408	3.239336015	0.001348809	0.033976898
DNMT1	0.188062913	10.74779775	3.144537123	0.001850203	0.040982364
CIDEB	0.188105027	7.426238932	4.550379399	8.12E-06	0.001550972
LOC4420	0.188195699	6.796795956	5.710819562	2.97E-08	2.33E-05
RPL15	0.188519443	10.5397774	3.287474113	0.001145555	0.030922952
ZFP3	0.188807812	7.198073131	4.179602798	3.95E-05	0.004195649
NDUFB11	0.188926825	9.790550053	3.973109189	9.12E-05	0.007105245
ITGB7	0.18933969	7.636280921	3.086165591	0.002239431	0.046027805
PLCL2	0.189978079	7.311568239	3.110111359	0.002071429	0.0442738
RORB	0.190675352	6.604150767	4.01106275	7.84E-05	0.006380456
PTTG1	0.190879377	9.464660164	3.226507243	0.001408364	0.034809879
ALG6	0.191083658	8.310610163	4.824343275	2.36E-06	0.000681397
NOS2A	0.191085096	6.665330588	4.141863602	4.62E-05	0.00460036
KRT86	0.191228213	6.770029199	3.894915722	0.000124039	0.008492826
FLJ20273	0.191623652	7.881579065	3.551367767	0.000452393	0.018156921
C9orf142	0.191927153	8.83139299	3.774621578	0.000197244	0.011257239
ISOC1	0.192060773	8.275757421	3.558590789	0.000440682	0.01793168
UPB1	0.192781846	6.501281846	4.925164425	1.47E-06	0.000462798
TMEM51	0.193346911	9.049159925	3.183160972	0.001628099	0.037896407
CBX7	0.193435811	7.780827717	3.109568569	0.002075105	0.0442738
DNAJC9	0.194622861	9.559691416	4.512529514	9.59E-06	0.001740999
PODXL2	0.19566461	6.971287675	3.376537807	0.000842552	0.025983148
PER3	0.195924283	7.310939445	3.886815049	0.000128021	0.008550166
HOOK1	0.1960808	7.951792669	3.302090736	0.001089717	0.030141967
ZADH2	0.197066272	7.446763003	5.127461016	5.63E-07	0.000220728
LSM3	0.197550003	9.032205662	4.359428875	1.86E-05	0.002652281
FRAT2	0.197643193	8.388228897	3.471553735	0.000602775	0.021536204
PSIP1	0.197668461	7.272072366	5.039092626	8.60E-07	0.000299964
PKIA	0.198053806	7.057043601	3.74048029	0.000224524	0.012181382
FAM26F	0.198292382	7.261212004	3.071686993	0.002347011	0.047443315
PTMA	0.198334298	10.55521999	4.05683651	6.52E-05	0.005644101
LOC6432	0.200096834	10.24831224	3.227175315	0.001405203	0.034809879
TXLNA	0.200103998	9.890133083	4.857131759	2.03E-06	0.000609525
TM7SF3	0.200115681	8.293105741	3.066758618	0.002384703	0.047829454
NDUFA3	0.200423243	8.993524941	4.446808818	1.28E-05	0.002062683
CASP2	0.200475501	8.681027377	5.289194205	2.55E-07	0.000133386
MEI1	0.200976891	6.573557261	8.05028543	2.69E-14	6.56E-11
LOC3885	0.200997519	8.750376173	3.08726366	0.00223146	0.045909987
CCDC34	0.202584017	8.280768542	3.233517427	0.001375525	0.03434593

RNASEH2	0.202852182	7.900529206	4.343774	1.99E-05	0.002749869
C6orf192	0.20311075	8.218114466	3.662392659	0.000300892	0.014442048
WDR6	0.204305875	9.284275188	3.484753974	0.000575038	0.020811935
SLFN13	0.204530125	6.885524578	4.652424796	5.15E-06	0.00119225
ZNF683	0.204931674	6.775623726	4.415158883	1.46E-05	0.00228111
LOC6539	0.207488952	6.533097718	5.062676157	7.68E-07	0.000276767
BCKDHA	0.207761853	8.440437497	4.691843174	4.32E-06	0.00107776
SPATA18	0.209617495	6.95017792	4.14204668	4.61E-05	0.00460036
SLC44A2	0.210073786	9.757416072	3.604430476	0.000372744	0.01641343
CD96	0.210387589	7.181675173	3.296443048	0.001110985	0.030469592
HLTF	0.211183862	7.902424154	3.544520704	0.000463763	0.018427252
LOC6527	0.211578692	7.522611794	3.858448569	0.00014294	0.008999514
ADD3	0.212066171	8.562080689	3.084047278	0.002254881	0.046261911
WDR34	0.212575032	7.569500294	5.144367432	5.18E-07	0.000207078
ASF1B	0.21363277	7.809780173	3.632787057	0.000335784	0.015664915
DENND2I	0.213777418	8.020001444	3.487294266	0.000569839	0.020730252
INA	0.214213124	6.631407999	4.097606926	5.53E-05	0.005043643
C9orf45	0.214473138	7.275321	4.141098037	4.63E-05	0.00460036
LIG1	0.214562093	7.87531346	4.654706492	5.10E-06	0.00119225
RINL	0.215510562	7.049098264	4.556715618	7.89E-06	0.001540664
DEK	0.215983498	10.04951766	3.363747086	0.000880909	0.026661454
LOC6473	0.216277754	9.201033578	4.984618205	1.11E-06	0.0003654
GPX4	0.216548175	9.788838346	3.592129152	0.000389939	0.016734619
LOC3412	0.216646988	6.997603999	4.012514965	7.79E-05	0.0063791
NEURL1E	0.217033459	8.304392589	4.273945844	2.67E-05	0.003205205
C18orf55	0.21719994	8.822169355	4.555865413	7.92E-06	0.001540664
CKLF	0.218952855	9.056147115	4.007463482	7.95E-05	0.006448959
HIST1H4	0.219018012	11.83208315	3.765881944	0.000203912	0.011488624
INPP5D	0.219060748	7.259888203	3.480556473	0.000583725	0.02099214
MEIS1	0.220248411	7.852671521	4.065039153	6.31E-05	0.005525625
MCM5	0.220496829	8.277101326	3.970750983	9.20E-05	0.007127413
NASP	0.220606068	7.75200577	4.213895596	3.43E-05	0.003768943
SKAP1	0.222363874	7.003273405	3.906443398	0.000118575	0.008253561
FAM108C	0.222405139	10.26337527	3.36392127	0.000880376	0.026661454
ARHGEF1	0.222543633	8.099684589	3.212014974	0.001478569	0.035845051
RBM47	0.222558552	8.890198071	3.27926809	0.001178058	0.031567639
FANCG	0.222934105	8.368199359	4.523117993	9.15E-06	0.001704231
NOS2	0.222962115	6.69380078	4.92899056	1.45E-06	0.000461153
MCM3	0.223243464	9.758896465	3.575095296	0.000414982	0.017408967
MAP4K1	0.225078193	7.189477437	3.198351305	0.001547717	0.036805169
ETV6	0.225252548	8.939635478	3.329680485	0.000991156	0.028420089
NOTCH1	0.225585328	8.800981734	3.568050508	0.000425775	0.017618741
CHAF1B	0.227184031	7.506941778	5.0118661	9.79E-07	0.000331281
PPM1M	0.2273484	8.141289312	3.887627528	0.000127616	0.008550166
TYMS	0.228578134	8.662862334	3.155445446	0.001784794	0.040152217
BARD1	0.229662742	8.00437908	4.483553551	1.09E-05	0.001912896
C10orf6	0.230396134	8.295030351	3.301493937	0.001091947	0.030141967
LOC7305	0.231078488	11.00079897	4.025861786	7.39E-05	0.006207969
PTTG3P	0.231623543	9.370699187	3.495305047	0.000553732	0.020393018
SAP30	0.232839378	8.107690858	4.350634099	1.93E-05	0.002718304
CENPK	0.235020942	7.641051239	4.099328511	5.49E-05	0.005043643

TMEM173	0.235488947	9.170789869	3.398947408	0.000779081	0.025095509
P8	0.235733637	7.318214753	3.458320636	0.000631833	0.02220925
RALGPS1	0.235937444	7.347316039	3.894851268	0.00012407	0.008492826
MGMT	0.236798106	8.460323989	3.306509205	0.001073343	0.029967673
GZMH	0.237061724	7.121346856	3.094657576	0.002178465	0.045177126
ACAP1	0.239749197	7.223141508	3.332671578	0.000980985	0.028399438
APOBEC3	0.240028966	7.032227828	4.139025172	4.67E-05	0.00460036
AK3	0.241990187	9.471766947	4.173225992	4.06E-05	0.004215145
KRT7	0.242204776	6.88070823	3.106736546	0.002094381	0.044505244
TRMT5	0.243216354	10.49028118	4.174368014	4.04E-05	0.004215145
SERBP1	0.244763677	8.926384149	5.593642863	5.47E-08	3.54E-05
PPFIBP2	0.246476129	8.824915457	3.3547995	0.000908702	0.027239992
LOC14891	0.247873009	10.09094898	4.299665816	2.40E-05	0.003042493
C5	0.248716899	7.330211722	4.763484787	3.12E-06	0.000845536
HADH	0.249925245	9.496022935	4.013420571	7.77E-05	0.0063791
SLC25A23	0.25044892	8.726545998	3.387898358	0.000809796	0.0254881
TYK2	0.250777831	9.458096595	3.729235665	0.000234262	0.012524205
CD6	0.25181703	7.18863374	3.408073485	0.000754535	0.024708502
PVRIG	0.253173743	6.851279324	4.180132021	3.95E-05	0.004195649
PHPT1	0.253479723	8.454642471	4.409477932	1.50E-05	0.002304779
ABCA3	0.254697852	6.882996053	3.085898149	0.002241376	0.046027805
EYA2	0.254983755	7.642611052	3.125725064	0.001968231	0.042650824
NUPR1	0.255842631	7.67835434	3.37837129	0.000837183	0.025945596
HCST	0.257487791	7.838830001	3.127650225	0.001955841	0.042508095
C14orf106	0.258125577	8.675411392	5.007328353	1.00E-06	0.000333073
LOC40098	0.258518254	8.459942591	3.437923529	0.000679202	0.023172955
SIM2	0.263224129	7.001361945	5.155116648	4.92E-07	0.000207078
MCM6	0.264420153	9.97265693	4.295139783	2.44E-05	0.003048284
MAP7D2	0.266881705	6.699647808	4.426684147	1.39E-05	0.002201713
TNFAIP2	0.267123134	7.393869202	4.377283593	1.72E-05	0.002522981
FOXRED2	0.267384728	7.297103764	5.793601619	1.92E-08	1.69E-05
C6orf173	0.268691946	9.234646204	3.55791008	0.000441773	0.017942861
ZCWPW1	0.269172501	7.532555189	4.278776658	2.62E-05	0.003175496
SMC1B	0.271873861	6.523196231	9.422424019	2.12E-18	1.27E-14
LOC73245	0.272406763	7.834774214	3.991699776	8.47E-05	0.006792045
GGA2	0.274982183	8.347333997	4.566382207	7.56E-06	0.001501482
CD8A	0.27544722	7.327771043	3.46524203	0.000616475	0.021989942
FAM3B	0.275850461	7.069548397	3.567140907	0.000427187	0.017643328
GCHFR	0.278569376	7.426869409	5.147185935	5.11E-07	0.000207078
RASEF	0.278615465	7.264929298	4.098280276	5.52E-05	0.005043643
CORO1A	0.280145738	7.588904987	3.207710349	0.001500038	0.036220152
C11orf92	0.281649946	6.765459039	3.261210112	0.001252623	0.032614967
CXCL17	0.28244706	7.345056723	3.240870197	0.001341846	0.033976898
TCP11	0.286424511	6.519741991	8.272830094	6.14E-15	1.69E-11
CDC7	0.287759137	7.986191761	5.070918499	7.39E-07	0.000270483
NUSAP1	0.289669537	9.102846714	4.336558556	2.05E-05	0.002779156
EVI1	0.289771019	7.912749354	3.276594617	0.001188831	0.031707744
CDC42EP	0.289862045	9.939207861	3.634673629	0.000333451	0.015589202
LTF	0.290009046	6.942191166	3.148196916	0.001828015	0.040613722
C1orf59	0.290651559	8.496067951	3.712380363	0.00024961	0.012996865
E2F2	0.291052106	8.670384206	4.222271318	3.31E-05	0.00371425

ATP6V0E	0.291355008	7.736050255	3.599167764	0.000380011	0.016469393
CYP2E1	0.293332247	7.379543435	3.05140076	0.002505762	0.049203842
NKG7	0.293399123	7.539672348	3.211386337	0.001481686	0.03585583
TJP3	0.294110433	7.226703041	3.601125749	0.000377292	0.016416306
NUP210	0.296527595	7.485244193	3.988529819	8.58E-05	0.006838213
TDRD9	0.296775313	6.642948368	5.352342381	1.86E-07	0.000101555
LOC2842	0.30017749	6.887654721	4.501435897	1.01E-05	0.00179794
PAFAH1E	0.304355529	9.375752041	4.518300826	9.35E-06	0.00172602
TMPRSS2	0.304972579	7.435564262	3.160475377	0.001755359	0.039681589
CELSR3	0.305941657	7.433345025	4.269312625	2.72E-05	0.003250407
EOMES	0.307981123	7.228585362	4.138334887	4.69E-05	0.00460036
PTPRCAP	0.310140095	7.323845408	3.602962947	0.000374757	0.016416306
VCAM1	0.310643922	7.720042061	3.309065659	0.001063974	0.02974388
GVIN1	0.314299327	7.706020558	3.338029924	0.000963006	0.028101093
MCM2	0.316019796	8.448632323	3.899994707	0.000121603	0.008402443
CD2	0.316894337	8.085756524	3.368669282	0.00086596	0.026464159
MLF1IP	0.317665705	7.866121797	4.703216221	4.10E-06	0.001047542
LOC6067	0.318285517	7.550565115	3.509867343	0.000525538	0.019822138
PDXK	0.319299406	9.367085522	5.264758129	2.88E-07	0.000143664
GZMK	0.321306228	7.329086891	3.420973738	0.000721068	0.023994813
HMGB2	0.322339617	9.548558325	4.800054984	2.64E-06	0.000723965
LOC3893	0.322966388	7.085297374	4.306776166	2.32E-05	0.002988454
C16orf75	0.323718804	8.452942442	4.181250402	3.93E-05	0.004195649
CYP4X1	0.32515496	7.67947735	3.267678035	0.001225426	0.032169986
TAF7L	0.327707282	6.527838467	7.534762287	7.49E-13	1.18E-09
LOC1001	0.328100578	8.879047533	3.682757802	0.000278905	0.014064539
COL9A2	0.32896173	7.507549406	3.373053742	0.000852843	0.026136005
ALOX5AI	0.330030909	9.459202893	3.187862117	0.001602818	0.037582513
RASIP1	0.334421621	8.422023408	3.051074289	0.002508395	0.04921158
CD247	0.340625351	8.294889091	3.183668619	0.001625351	0.037872573
RNF212	0.342903107	6.859697293	5.273857145	2.75E-07	0.000140549
NEFH	0.345478225	6.746220656	7.313547209	3.00E-12	4.12E-09
CD52	0.34548223	7.648098148	3.599699062	0.000379271	0.016469393
TBC1D10	0.345948589	7.454322765	3.634867176	0.000333213	0.015589202
C2orf55	0.348990841	7.68451227	4.153232559	4.41E-05	0.004445734
RPA2	0.35557749	9.589856312	7.059624177	1.42E-11	1.84E-08
TCEA3	0.357826699	9.02114652	3.769520645	0.00020111	0.011411205
CD48	0.360091968	8.014663681	3.066135603	0.002389507	0.047829454
LTB	0.361126336	7.856861092	3.331219703	0.00098591	0.028420089
KLRB1	0.376724032	7.676875444	3.606855477	0.000369439	0.016366316
APOC1	0.380721443	7.861142557	3.374138557	0.000849626	0.026073793
CD3D	0.380902514	8.222550182	3.384597259	0.000819189	0.025641096
LMO4	0.389339763	9.31652491	4.592930917	6.72E-06	0.001419142
CLDN10	0.393511025	7.293577282	3.309145305	0.001063683	0.02974388
UCP2	0.394426046	7.962266698	3.938866058	0.000104403	0.007621426
ARHGAP	0.395494019	7.679199252	4.755857036	3.23E-06	0.000864839
LOC3884	0.397616569	6.548610368	9.262599982	6.64E-18	2.43E-14
MGC4236	0.398700611	8.076348625	4.295620807	2.44E-05	0.003048284
CDKN2A	0.422898764	6.832198548	6.495388532	3.99E-10	4.39E-07
FOXA1	0.427651034	8.011523395	3.058896458	0.002445987	0.048479641
LOC6498	0.430117963	9.209038226	4.229320517	3.22E-05	0.00368197

LOC1001:	0.430809515	8.885914234	3.094527028	0.002179391	0.045177126
STAG3	0.432415186	6.753401424	9.040819172	3.18E-17	9.97E-14
CLDN7	0.438366422	8.680034857	4.407088799	1.52E-05	0.002304779
TCAM1	0.441669017	6.812536327	9.361672938	3.28E-18	1.44E-14
CBS	0.457025035	8.000089323	4.342548757	2.00E-05	0.002749869
PLAC8	0.457611932	7.84368786	3.453893472	0.000641844	0.022315256
YBX2	0.45947695	7.162969943	6.216348644	1.93E-09	1.93E-06
KLHL35	0.464211451	6.988755685	7.979373282	4.28E-14	8.55E-11
GABRP	0.491825008	7.851979391	3.171420566	0.001692853	0.038787338
ZNF541	0.51273037	6.670311946	10.50509352	7.49E-22	1.65E-17
UBD	0.552878412	8.318983367	3.450463137	0.000649703	0.022446409
CALML5	0.606598806	8.751092566	3.675228793	0.000286851	0.014260115
SYCP2	0.637015623	6.902684651	9.771105666	1.71E-19	1.88E-15
KCNS1	0.646040837	7.000397146	7.589436896	5.30E-13	8.95E-10
KRT19	0.759971762	10.1046093	3.505478845	0.00053389	0.02005909
C4orf7	0.896707139	7.806618507	4.241348945	3.06E-05	0.003557398

5858 cohort.
B
2.783771283
-0.363994114
3.06123948
3.040310859
2.510510249
0.632193104
-1.288576695
-0.77132369
0.431931537
-0.855290148
1.267142939
0.913337371
-1.533441108
-0.885296126
-1.728513905
-1.820956394
2.038329802
5.616176342
3.630856937
1.883996031
2.944747088
-0.178905365
-1.438098674
-0.402716745
3.057643645
3.618781573
-0.878639892
1.972382533
-1.374367808
0.583012374
1.709895076
-1.016627009
-0.313781477
-1.374760349
5.959352618
0.064489273
-0.311596586
-0.561768841
3.366105175
2.567479994
2.772139814
1.713858875
-1.388281756
-0.866166451
3.802422923
1.007816877
-0.846679813
-0.456612991
-1.394773669

0.745874327
1.034785166
-0.54001397
-1.883041246
-0.434033932
-1.36558483
0.349341706
5.327089329
-0.819802752
-1.727366403
-1.38103481
-1.219133296
-0.534750875
-1.32605286
2.118361312
0.234999349
-1.220939196
-1.888960895
-0.766364784
1.869722432
-1.538918273
0.103836253
-1.684513725
-1.074684036
-0.762212567
-0.336640541
-1.469765813
0.729464644
0.229902751
0.487173064
-1.4822001
-0.14409991
3.519188842
1.912063328
-1.720117883
0.438943226
1.730632
-1.224478931
-0.973533988
0.333061778
1.006830452
-1.337278862
-0.174458655
-0.56585825
-0.896725121
-1.80762431
-0.974563292
1.787221867
3.263629434
0.471940569
-1.310409538

-1.051560223
3.465896328
-1.641868676
4.121363966
-0.753078479
-1.309543766
-0.463367957
-0.736220517
-0.637761983
-1.556980426
-1.014255311
1.449291288
0.431054963
-0.107729566
0.030473757
0.825991555
-1.516934076
1.317489711
0.085434593
-0.908202507
0.577718588
0.289227155
1.170032747
-1.913124916
0.374023904
-1.830582289
1.208904553
-0.909305961
-1.551725873
2.368116898
-0.634636987
-1.481793179
-0.810950941
0.923528058
7.522331114
-1.158943239
-1.741583133
-0.20038037
0.026948066
-1.366907528
1.489539683
-1.63294668
-0.154414999
5.607879645
-0.383602821
-1.266062843
0.780453581
0.278876101
0.77628059
0.278792673
0.355362949

1.34863952
-0.033924508
-0.839083518
0.682451195
0.49762236
-1.835133166
-1.474125734
-0.831650759
-1.688066852
-1.193639996
4.033455903
0.294455186
0.727171576
-0.138190714
-1.381522386
0.277402343
-1.437382353
-1.32914439
0.168804562
4.409275395
-1.574819202
-1.528258446
1.524307438
-1.060393764
-1.323259051
-1.314843001
0.311183358
-0.55014819
-1.487581907
0.181380285
0.761799973
0.481093888
0.201730518
-1.471486276
1.484312691
0.976719648
0.190266246
-0.86087812
-1.446560883
-0.063690667
1.573607706
-1.218255189
2.378695851
-0.71092301
0.948444335
0.149896653
-0.439662311
-0.34156671
1.589146228
-0.445873877
0.370866091

1.627098085
0.451472618
-0.241778217
-1.878791948
-1.710029858
-0.982230875
-0.274993288
1.275193208
-0.15927053
2.296050644
-0.143463828
-1.546420429
-1.142738973
0.594583502
-1.370508648
1.105793393
-1.129824728
-0.732506974
-1.102252184
-1.890251575
-1.812405879
-0.779271636
-0.030846684
-0.34800372
0.051338852
-1.452222888
0.623675714
-1.538618473
-1.59002768
3.074106649
-0.500862305
-0.862259109
-0.74396379
3.313489625
-0.296766763
-1.835027082
-0.4447141
-1.15169184
0.411859723
-1.819451831
0.710516094
-0.234014846
1.010768712
3.711413546
0.151014041
-1.595540576
0.476378442
-0.874026367
0.994399327
-1.026445124
-1.892789506

0.025171481
-1.503325681
-0.477835018
-0.66189715
-1.246023636
-1.14110347
-1.535105171
-0.380176031
-0.82912574
-1.3357809
-0.443515055
0.937660737
-1.706334908
-1.160095318
-1.336032841
-1.113968085
-0.590399316
1.821713636
-1.251091294
-0.343599872
0.995088482
-1.844763132
-1.503905183
-1.488083975
0.042983863
-1.092405822
-0.255951984
-1.868315781
-0.541223019
3.513454246
-1.748255759
0.830903637
-0.059746678
-1.747085168
2.826224979
-1.637732965
-1.474110622
-1.41097379
-1.758409421
-0.994763033
-0.650345662
-1.757837308
-1.809898187
-0.105717025
-0.844291086
-1.897786781
-1.842927268
-1.774225933
-1.195348685
1.089757185
0.735283691

-0.847648165
3.607745794
-1.873368355
0.475371386
0.663420596
-1.227245933
-1.30210728
-1.74667736
-1.896646003
-0.439437238
0.014611895
-1.27021039
-1.411375883
-0.26743194
1.531538739
-1.603943633
-0.731703826
-0.708341534
0.588997985
0.033092914
-1.807801741
0.602397996
-1.663265701
-1.911009176
-0.748706754
3.964604121
-1.594625528
-0.853281994
-0.677925363
-1.267197001
-1.876570407
-1.840851577
-1.809167205
-0.946477233
-0.110046388
-1.652626769
-0.996863121
1.644958529
0.075858674
-1.876578995
-1.50890979
-0.700895283
-0.39405873
-1.754207051
-1.44014045
-1.286647179
-1.717317619
-1.73947668
-0.659133058
-1.546699253
-0.915503207

-1.531194517
-0.768943758
-1.737395868
-0.820421762
0.912394768
-0.009742182
-1.462709674
-1.053969768
2.222515422
-1.733945307
-0.942701178
-1.292123452
-0.364862894
-0.826778061
-1.099932436
-1.891003026
-1.147102848
-1.257236464
-1.055495872
-0.638230307
1.497657188
-0.63358407
-1.055039669
-0.496076087
-1.898735763
-0.10818055
-1.82426881
-1.797155862
-1.604121699
0.072897507
1.063532959
-0.714114806
-1.849207045
-1.305933285
-1.426671934
-0.867732867
-1.023952178
-0.803548481
-1.766941673
-0.649926196
-1.734112301
-0.429272716
-1.09689613
-0.433437686
-0.998883654
-0.98407201
-1.554540274
-0.895057867
-0.999723779
-0.43402892
-0.166758844

-1.623695411
-0.899140915
-1.607950485
-1.871994821
-0.659634956
-0.115115387
-1.758208849
0.007930749
-1.851469088
-1.830475251
-0.698318142
-0.499722891
-0.482191896
-0.418729745
-0.542377448
1.420238582
-0.529352219
-0.667481283
-1.541151189
2.994711449
-1.230747654
-1.213700699
-1.226913009
-1.404455659
-0.866396231
-0.346912089
-1.318654458
-1.131640043
-1.292557166
0.836753558
-1.516597814
-1.178155998
-1.805750291
5.046143867
-1.76887452
2.982623067
-1.436386151
-0.370631463
-1.093955106
2.73913417
-1.261251643
-1.740357972
-0.235249497
0.038728372
2.910694523
-1.586624866
-0.281180329
1.146868724
-0.016111144
0.53776851
-1.175981304

-1.394016169
-1.168163284
-1.546147023
-1.140566423
2.302410364
-1.588626928
-1.276654879
-1.261312994
-0.136941867
0.469631724
-1.021531446
-0.28576382
-1.076071502
-1.174690588
-1.523810127
8.886924844
-1.076588865
-1.331331661
-1.041603716
-0.427956382
-0.905286407
-1.896028649
3.753592502
-1.689107168
-0.962329193
-1.168289272
-1.336300276
-1.087606269
-1.442334618
-1.550411956
-0.994431525
-1.451767093
-1.388902856
-1.176948348
-1.296312063
-1.578523302
3.681311171
0.321982483
-1.334185973
-1.089391416
-1.507271238
-0.715252287
-1.818636609
-1.167681169
-0.314298231
1.986589649
-1.908924553
-1.164663401
-1.805388933
-0.83829126
-0.666087319

0.500058566
3.834281772
-1.718437092
7.376658415
0.581055762
0.220498382
-1.908041388
-1.846694872
-1.495877553
-0.520577288
-1.753217306
-1.85253112
14.48139245
-1.045733499
-0.878063624
-1.705743077
1.04455853
-1.742116442
-1.304352814
-1.72187174
-1.354369571
-1.719960063
0.397761049
-1.241382966
2.171374514
0.026294917
-0.984638214
1.350819926
1.822024318
-0.891742224
-1.651629877
-1.644800231
-1.666678762
0.812735311
0.08730834
1.614479285
-1.659766611
-0.834558344
0.048398573
-1.415510413
1.080045917
-1.815286559
0.738015478
2.44184432
0.963343921
-1.812744649
0.491053133
7.777500841
-1.583796303
6.417930689
1.036102055

0.356668177
-1.462100585
-1.465209936
-1.872939672
-1.042460499
0.904464396
3.418145328
-1.631000429
-1.677560259
4.585069268
-1.002607492
-0.111280986
0.994724609
-1.057206904
0.312875377
-0.090035073
-0.861883166
-1.797744713
-0.75123796
-0.232491242
-0.646518743
3.749419587
-0.359900966
2.390756824
-1.038825911
-1.84835307
1.441617435
2.055079891
-0.85090409
-1.651425309
3.011582602
-1.325111169
0.061280958
2.073142793
1.666160229
-1.115416896
1.29291769
3.873905154
2.471519458
0.956664251
0.147688152
1.204380938
1.543232707
-0.280824505
4.757992756
-0.873849988
-0.387138511
-1.299568452
-0.192812727
-0.379564621
2.340440706

-0.436096742
1.106309983
2.681143865
0.411645226
8.438453404
6.093675191
0.246183708
-1.521303319
-1.761929934
0.133421357
0.789297029
-0.189065926
4.108178155
0.682852129
0.715042851
2.724552286
0.638712464
-1.352411066
-1.593764011
-1.587963528
3.179111545
1.417684229
-0.792415226
8.29294441
-0.595369451
-0.263981226
-1.848817608
0.730831253
-0.942536031
-0.990723897
-1.428892109
-0.401418184
1.646189367
-0.762462
0.45815473
9.797668871
0.059458665
-0.81176523
0.509288108
1.152972816
-0.831803302
-1.839013578
2.644783177
2.534809
-0.177966823
-0.518304581
8.044211826
2.486610134
0.748100215
0.97146002
-1.528841667

-1.209733916
-1.760217868
-0.866319414
-1.780803236
-0.913555136
-1.774502067
0.754434958
-1.275376278
4.420678059
-0.632273422
-1.856985188
-1.049645942
-1.686548205
-1.057175653
-0.233949173
-0.271080086
2.094980597
-1.49923912
2.517288727
1.64647872
-1.660454289
-0.566150543
0.937240882
-0.512294366
-1.527139661
-1.58626568
-0.094617323
-0.113401191
2.447472865
-0.51711365
-1.848968539
1.135126046
-0.960130053
-0.778480979
-0.041765446
0.583763804
-1.181956153
0.225783638
0.07999975
0.463710033
-0.093803357
-1.545430858
3.147681375
-0.529459849
-1.622542931
5.770702689
-1.678589443
2.690151535
-1.905203618
-1.093301346
0.258493049

0.305446979
0.495821076
-0.92616267
-0.7585326
1.271366008
0.113612918
-0.12664368
-0.97292429
0.135210569
-1.771069788
-1.412762395
8.671365018
1.310163954
-1.337263196
-0.687367734
-1.143581286
-0.647632186
6.255012359
-1.013495633
-1.037601639
-0.792811973
2.970463745
0.025743872
-0.180172252
2.057596915
-1.629723116
21.5542426
-1.434523227
-1.207497814
-0.792692256
1.061745717
0.387853249
-1.740560314
-1.36534196
1.565873044
-1.493008192
0.733532505
-1.229393855
1.000254855
2.275109581
-0.731056488
2.469143887
-1.761063484
17.6864277
-1.84501337
-1.376912218
-0.295642914
0.094199662
-1.133468048
-0.565918352
1.172383092

-1.734909978
0.39159352
1.711452277
-0.14606663
-0.438393927
2.146403836
-1.270258859
-1.898115228
-1.241114757
-1.332028314
0.22267311
2.257784799
2.039850084
-0.085692765
-1.414977865
0.196709969
6.366515793
-0.525747799
0.423018709
-0.995982263
0.975491705
-1.442185547
-0.939368164
20.96963579
0.294295916
-1.865266328
-1.36338617
-1.630402994
-0.333502728
9.105248711
-0.788510066
-1.719474313
2.942950751
-0.429929021
3.452208754
-0.22039953
2.428012576
5.949648306
-1.641587276
-0.25055824
-1.166871012
1.493951179
12.127498
-0.080620623
1.890225535
-1.591096425
-1.673342147
1.074934832
-0.888553349
7.459737229
-1.226034888

-0.314795962
-0.447051049
3.868994125
-0.755123055
-0.050528322
-1.339307284
-1.234530361
0.775568976
2.596924967
-0.982090752
-0.226399208
0.152673344
-0.963932386
-1.070994782
1.623708862
2.174812315
-1.908972472
-1.080644229
-1.746274905
0.732715785
5.531330271
6.286289601
-1.779688644
-0.522694911
-1.614485314
-0.207861367
4.671809925
1.725196777
1.862132419
12.95502123
1.144740071
1.791398032
0.943725904
-1.650006859
-1.470469843
0.217198109
-0.517120821
-1.847112649
-1.658756223
0.713587256
-0.346405082
-1.431839452
-1.13361128
-1.883840771
-1.247476323
3.866126887
2.021018834
3.460148239
-0.671053554
-1.670635413
0.809675518

-0.140199143
0.352072132
0.814588071
1.602572811
-1.191272159
6.077841667
-1.467997449
-0.99830099
-1.158212876
-0.651184465
-1.34944262
-0.403551429
1.83334966
-0.655692136
0.86606683
-1.401031915
8.421685594
-1.683605858
-1.437546301
-1.578950182
1.260890378
-0.269252299
-0.137374818
0.250777389
0.548153267
-0.804059616
-0.599694547
0.046762682
1.390100012
-0.16845424
-0.724425168
-0.939035269
0.876288623
0.829261697
-0.319592696
2.910447051
4.517131737
-0.098943702
-1.289907564
2.297137735
3.612285863
0.29704312
0.06624367
-0.554320608
1.702841945
7.156393309
-1.497244919
4.46437456
2.587763538
-1.905318431
-0.567418343

-1.554440279
0.726359729
-1.232412214
8.287901657
3.203586887
0.166629743
-0.403384316
-1.747650212
-1.387153014
-0.137135827
30.71947911
6.867419989
-1.33243309
-1.61573821
3.355086951
8.609388039
-1.185601793
1.888127698
1.118380398
-1.786300881
-1.716689744
1.257281823
-1.371226472
4.507054208
1.744890503
0.835902501
-0.345474289
0.411109886
-0.321649057
4.945168584
-1.501250724
-1.718273186
3.20042976
-0.908692337
0.806923676
-1.140623465
5.846768667
2.586163654
-0.605811394
5.449257535
0.292720879
-1.828148062
1.426353974
-1.369209771
4.648707461
-1.842350689
2.934516878
6.588835013
21.80500471
-1.783119693
-1.350045728

2.524383097
0.025579428
-0.563126582
3.777520686
2.807652447
5.554790431
3.942822641
1.745582621
-0.169423807
-1.158024312
-0.368018854
0.705870828
-1.792434818
5.923462669
-0.074374343
-0.554895265
1.578367659
1.741996613
3.78705689
3.381084787
-0.948877741
5.207042277
-0.210447493
1.262619784
2.251157122
3.377594562
1.244059215
0.380712303
-0.57671579
1.456830275
1.109788463
2.019268425
0.877233389
-0.948331434
-1.414879204
-1.210772918
3.243583915
4.961943103
-0.267043981
-1.455869808
-1.055224422
-0.290379647
5.327926485
0.809827763
-1.583535287
3.082778201
-1.14246353
1.311759089
-0.528902131
2.551432225
1.584816042

-0.837950045
-0.648454244
0.835671716
-1.126990819
-1.761671594
-1.045926825
1.734163383
1.863845074
-1.726530683
1.868191466
8.035599916
-0.976905819
2.351348634
4.246260143
1.265949418
-0.872883059
0.253940179
-0.809018294
1.890144415
2.784963622
-1.787075522
-1.671031206
-0.902920521
-1.665386844
5.307757269
-0.713891646
5.972332185
2.333679773
2.853759723
2.656859894
9.020313661
-0.323896259
2.26993564
30.80013951
1.186271805
3.42080666
-0.626168997
-0.293389635
5.936268484
1.58088948
-1.42781037
-1.265959882
-1.327784316
23.21031103
5.591769018
2.495972683
-1.218961014
-0.068026793
-1.604945495
0.196005779
2.051440445

-0.186989963
-1.886472986
-1.416768638
-0.180457211
1.174675531
6.883612728
3.155309595
3.223940473
-1.568651418
2.23316401
1.731555604
-0.174324539
-1.119095538
-1.029251633
0.854099112
-0.933429912
3.990734549
-0.481511853
6.475522118
-0.768000549
4.40264044
2.379138805
1.894406978
-1.246225532
18.63956235
0.09476045
-0.919652287
-1.487227462
-1.887408681
-1.499737362
6.517665886
17.32204864
-0.185217622
-0.067375416
1.787921123
15.84169588
0.393360504
-1.844144587
-1.050440831
-0.161321699
-0.916240857
-0.883299713
3.530268774
-1.118849471
0.994062123
4.213769032
29.71447692
2.335556844
12.68164941
-1.864964002
2.078560297

-1.762050697
28.22336572
2.775429309
30.38639547
2.519555893
-0.66268735
11.18865662
21.36199957
-1.536188101
38.37374252
-0.673704268
0.06914389
33.19925639
18.96907195
-0.49581227
2.124927322
