# Supplementary material for: HPV-Related Prognostic Signature Predicts Survival in Head and Neck Squamous Cell Carcinoma
Source: J Oncol. 2022 Nov 15;2022:7357566. doi: 10.1155/2022/7357566 (PMC9681561; doi:10.1155/2022/7357566)
Supplement: Supplementary Materials — Supplemental Table 1: HPV-associated signatures with significant differences from the GSE65858 cohort. Supplemental Table 2: clinical information of the GEO cohort. Supplemental Table 3: clinical information of TCGA cohort. Supplemental Figure 1: ROC curves of the prognostic signature (A); the relationship between the prognostic signature and HPV status (B). [file 7357566.f1.zip › Supplemental Table 2.pdf]

Supplementary Table 2: Clinical information of GEO cohort.

**Variable**	**Case, *n***
Age	
>65y	86
≤65y	183
Gender	
Male	222
Female	47
Status	
Alive	176
Dead	93
TNM Stage	
I	18
II	37
III	37
IV	177
T classification	
T1	35
T2	80
T3	58
T4	96
M classification	
M0	262
M1	7
N classification	
N0	94
N1	32
N2	131
N3	12
Smoking classification	
Yes	221
No	48
Drinking classification	
Yes	238
No	31
HPV status	
HPV16	60
Negative	196
Other HPV	13
