# Supplementary material for: HPV-Related Prognostic Signature Predicts Survival in Head and Neck Squamous Cell Carcinoma
Source: J Oncol. 2022 Nov 15;2022:7357566. doi: 10.1155/2022/7357566 (PMC9681561; doi:10.1155/2022/7357566)
Supplement: Supplementary Materials — Supplemental Table 1: HPV-associated signatures with significant differences from the GSE65858 cohort. Supplemental Table 2: clinical information of the GEO cohort. Supplemental Table 3: clinical information of TCGA cohort. Supplemental Figure 1: ROC curves of the prognostic signature (A); the relationship between the prognostic signature and HPV status (B). [file 7357566.f1.zip › Supplemental Table 3.pdf]

Supplementary Table 3: Clinical information of TCGA cohort.

Variable	Case, n
Age	
>65y	174
≤65y	324
Gender	
Male	365
Female	133
Grade	
G1	61
G2	298
G3	118
G4	2
unknow	19
Status	
Alive	281
Dead	217
TNM Stage	
I	25
II	69
III	78
IV	258
unknow	68
T classification	
T1	45
T2	131
T3	96
T4	170
unknow	56
M classification	
M0	184
M1	1
unknow	313
N classification	
N0	169
N1	65
N2	164
N3	7
unknow	93
Smoking classification	
Yes	378
No	110
unknow	10
Drinking classification	
Yes	330
No	157
unknow	11
HPV status	
Positive-p16	30
Negative	79
Other HPV	3
