# Supplementary figures and images for: HPV-Related Prognostic Signature Predicts Survival in Head and Neck Squamous Cell Carcinoma
Source: J Oncol. 2022 Nov 15;2022:7357566. doi: 10.1155/2022/7357566 (PMC9681561; doi:10.1155/2022/7357566)

A

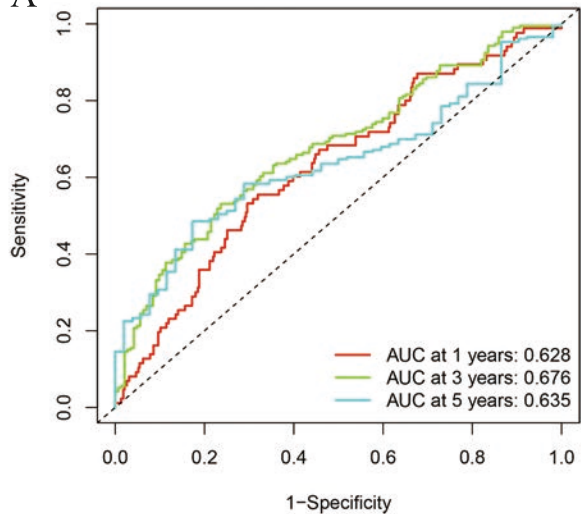

B

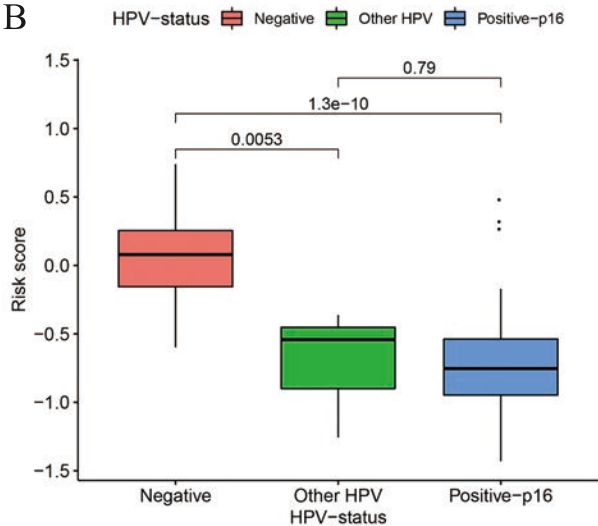

Supplement: Supplementary Materials — Supplemental Table 1: HPV-associated signatures with significant differences from the GSE65858 cohort. Supplemental Table 2: clinical information of the GEO cohort. Supplemental Table 3: clinical information of TCGA cohort. Supplemental Figure 1: ROC curves of the prognostic signature (A); the relationship between the prognostic signature and HPV status (B). [file 7357566.f1.zip › Supplemental Figure 1.pdf]
